# Intracellular Growth Is Dependent on Tyrosine Catabolism in the Dimorphic Fungal Pathogen *Penicillium marneffei*

**DOI:** 10.1371/journal.ppat.1004790

**Published:** 2015-03-26

**Authors:** Kylie J. Boyce, Alisha McLauchlan, Lena Schreider, Alex Andrianopoulos

**Affiliations:** 1 School of BioSciences, The University of Melbourne, Parkville, Australia; 2 South Australian Clinical Genetics Service, SA Pathology, Adelaide, Australia; University of Rochester, UNITED STATES

## Abstract

During infection, pathogens must utilise the available nutrient sources in order to grow while simultaneously evading or tolerating the host’s defence systems. Amino acids are an important nutritional source for pathogenic fungi and can be assimilated from host proteins to provide both carbon and nitrogen. The *hpdA* gene of the dimorphic fungus *Penicillium marneffei*, which encodes an enzyme which catalyses the second step of tyrosine catabolism, was identified as up-regulated in pathogenic yeast cells. As well as enabling the fungus to acquire carbon and nitrogen, tyrosine is also a precursor in the formation of two types of protective melanin; DOPA melanin and pyomelanin. Chemical inhibition of HpdA in *P*. *marneffei* inhibits *ex vivo* yeast cell production suggesting that tyrosine is a key nutrient source during infectious growth. The genes required for tyrosine catabolism, including *hpdA*, are located in a gene cluster and the expression of these genes is induced in the presence of tyrosine. A gene (*hmgR*) encoding a Zn(II)2-Cys6 binuclear cluster transcription factor is present within the cluster and is required for tyrosine induced expression and repression in the presence of a preferred nitrogen source. AreA, the GATA-type transcription factor which regulates the global response to limiting nitrogen conditions negatively regulates expression of cluster genes in the absence of tyrosine and is required for nitrogen metabolite repression. Deletion of the tyrosine catabolic genes in the cluster affects growth on tyrosine as either a nitrogen or carbon source and affects pyomelanin, but not DOPA melanin, production. In contrast to other genes of the tyrosine catabolic cluster, deletion of *hpdA* results in no growth within macrophages. This suggests that the ability to catabolise tyrosine is not required for macrophage infection and that HpdA has an additional novel role to that of tyrosine catabolism and pyomelanin production during growth in host cells.

## Author Summary

Fungi that infect humans are a major health problem, especially for those with compromised immune systems. Many fungal infections are extremely difficult to cure and if left untreated are fatal. For successful infection to occur, the fungal pathogen must be able to grow by acquiring and utilising the available nutrient sources within the host whilst evading or tolerating the host’s defence systems. Expression profiling in several pathogenic fungal species has revealed that genes required for tyrosine catabolism are induced specifically in the pathogenic cell type at 37°C. As well as enabling the fungus to acquire carbon and nitrogen intermediates from proteins within the host, tyrosine is also an important precursor in the formation of two different types of melanin, which protects cells against the host’s defence systems. This study shows that the ability to catabolise tyrosine and produce tyrosine derived melanin is not required for the initial stages of fungal infection. However, a novel role for *hpdA*, which encodes the enzyme which catalyses the second step of tyrosine catabolism, was identified during growth in host cells.

## Introduction

A number of pathogenic microbes reside within phagocytic cells of the host innate immune system, a strategy that minimizes exposure to the adaptive immune response but which requires subversion or escape from the cytotoxic capacity of innate immune cells. The acquisition of nutrients for growth is a key challenge faced by these intracellular pathogens, which have to scavenge nutrients from the relatively nutrient poor environment of the macrophage. Expression profiling studies using a number of intracellular pathogens has revealed distinct changes to metabolism upon phagocytosis, specifically for genes involved in carbon assimilation from host proteins and amino acids [1, 2, 3, 4]. Many fungi can readily assimilate amino acids as both carbon and nitrogen sources and proteins are relatively abundant in host cells. There is good evidence that amino acids are an important nutritional source for pathogenic fungi. For example, genes required for tyrosine catabolism are induced under infection conditions in the fungal pathogens *Paracoccidioides brasiliensis*, *Histoplasma capsulatum*, *Penicillium marneffei* (recently renamed *Talaromyces marneffei*) and *Aspergillus fumigatus*, suggesting that tyrosine may provide an important source of carbon and/or nitrogen during infectious growth [2, 4, 5, 6]. Tyrosine is catabolized via a pathway that is conserved across the kingdoms. Garrod originally identified this pathway in his classic work on inborn errors of metabolism in humans and the full pathway was uncovered by elegant studies in the model fungus *Aspergillus nidulans* [7, 8]. The catabolism of tyrosine provides the fungus with nitrogen from a transamination reaction with α-ketoglutarate to produce glutamate and carbon via production of fumarate and acetoacetate, which is further catabolised to acetyl-CoA, which feed into the TCA cycle [7].

As well as enabling the fungus to acquire carbon and nitrogen intermediates from proteins within the host, tyrosine is also an important precursor in the formation of two different types of melanin. Melanins are a large group of pigment macromolecules which although chemically diverse, display similar chemical properties and function to protect cells from environmental stress. Melanins are broadly classified into one of three groups: pheomelanins, eumelanins and allomelanins (pyomelanins and DHN-melanins) [9]. Tyrosine can be hydroxylated by tyrosinases and/or laccases to produce DOPA that can then be used for the formation of the eumelanin DOPA melanin (L-3, 4-dihydroxyphenylalanine melanin), while catabolism of tyrosine produces the pathway intermediate homogentisate that can be used to generate the allomelanin pyomelanin through oxidation and polymerization [10, 11]. The two most commonly identified melanins in fungi are DOPA-melanin and DHN-melanin (1,8-dihydroxynaphthalene melanin); the latter being synthesised from polyketides made from acetate precursors. Both DHN- and DOPA-melanin have been shown to protect fungal cells from RNS and ROS derived from host macrophages [12, 13, 14, 15]. Mutants which block the biosynthesis of DOPA-melanin in *Cryptococcus neoformans* and DHN-melanin in *A*. *fumigatus*, *P*. *marneffei* and *Wangiella dermatitidis* show reduced virulence in murine models of infection [12, 13, 14, 15, 16]. Melanisation has also been shown to influence phagocytosis, phagolysosomal maturation and the release of proinflammatory cytokines during infection [17, 18, 19, 15, 20, 21, 22].

A number of important human fungal pathogens are dimorphic and switch from a non-pathogenic multicellular hyphal form found outside the host to a unicellular yeast growth form during infection. Microarray-based expression profiling in *P*. *marneffei*, *P*. *brasiliensis* and *H*. *capsulatum*, to identify differential expression between the two growth types, has revealed that genes required for tyrosine catabolism are induced specifically in the pathogenic cell type at 37°C [2, 4, 6]. The melanin synthesized *ex vivo* by these pathogens has been previously postulated to be either or both of the two most commonly identified melanins, DHN-melanin and DOPA-melanin [23, 24, 25, 26]. However, this raises the possibility that these fungal pathogens may be producing the third type of melanin, pyomelanin, via the oxidation and polymerization of homogentisate produced during tyrosine catabolism [10, 11]. To investigate this possibility, and the role of tyrosine catabolism during infectious growth, the role of genes required for the catabolism of tyrosine was investigated in *P*. *marneffei*. The genes required for tyrosine catabolism are located within a conserved gene cluster. This study shows that the expression of genes within the cluster is both positively and negatively regulated by a gene (*hmgR*) encoding a C6 binuclear cluster DNA binding motif transcription factor, present within the cluster, in response to nitrogen source availability. The expression of tyrosine catabolic cluster genes is also under the regulation of the global GATA-type transcription factor AreA. Deletion of genes of the tyrosine catabolic cluster reveals the requirement of the cluster for growth on tyrosine as both a nitrogen and carbon source at both 25°C during hyphal growth and at 37°C during yeast growth. This study also shows that *P*. *marneffei* produces pyomelanin when grown on medium containing tyrosine at 37°C and this melanisation requires genes of the tyrosine catabolic cluster. Deletion of the genes of the tyrosine catabolic cluster, with the exception of *hpdA*, does not affect infectious growth in macrophages. This result suggests that tyrosine catabolism and pyomelanin formation are not required for the initial stages of infection, however, HpdA has an additional novel role during *ex vivo* infectious growth of *P*. *marneffei*.

## Results

### HpdA is essential for *ex vivo* yeast cell production during infection

**Fig 1:**
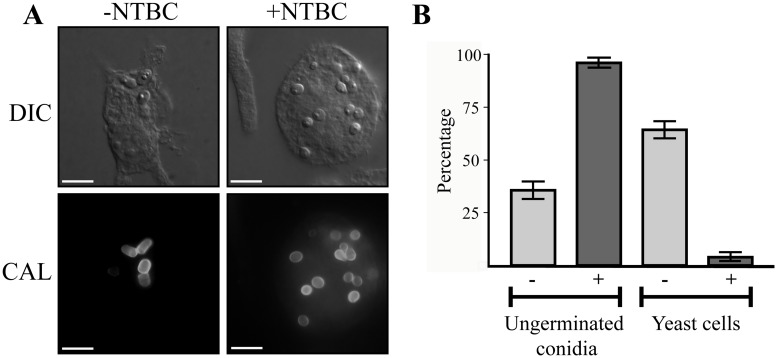
*hpdA* is essential for yeast cell production during *P*. *marneffei ex vivo* growth in macrophages. A. Macrophages infected with wildtype conidia without (-) or with (+) 400μg mL^-1^ of the HpdA inhibitor NTBC added to the macrophage media at the time of infection. After 24 hours, macrophages infected with wildtype conidia without NTBC added contain numerous yeast cells dividing by fission. In contrast, the addition of NTBC results in predominately ungerminated conidia remaining in macrophages 24 hours post-infection. B. Quantitation of the numbers of ungerminated conidia and yeast cells after 24 hours without (-) or with (+) NTBC.

Microarray-based expression profiling in *P*. *marneffei* revealed that *hpdA*, encoding 4-hydroxyphenylpyruvate dioxygenase which catalyses the conversion of 4-hydroxyphenylpyruvate to homogentisate during tyrosine catabolism, is induced specifically in the pathogenic cell type at 37°C [6]. To assess if *hpdA* is required for *ex vivo* yeast growth, wildtype conidia were used to infect murine J774 macrophages in the absence and presence of the 4-hydroxyphenylpyruvate dioxygenase (HpdA) chemical inhibitor NTBC (2-(2-nitro-4-trifluoromethylbenzoyl)-cyclohexane-1, 3-dione) and examined 24 hours post-infection [2, 27, 28]. After 24 hours, wildtype conidia within macrophages have completed isotropic expansion followed by elongation into a cylindrical yeast cell. Macrophages infected with wildtype conidia contain numerous yeast cells, some of which are dividing by fission (Fig. 1A and B). In contrast, predominately ungerminated conidia were observed in macrophages in the presence of NTBC (Fig. 1A and B). This suggests that tyrosine catabolism plays an important role during *ex vivo* yeast cell production in *P*. *marneffei*.

### Genes required for tyrosine catabolism are located in a conserved gene cluster

**Fig 2:**
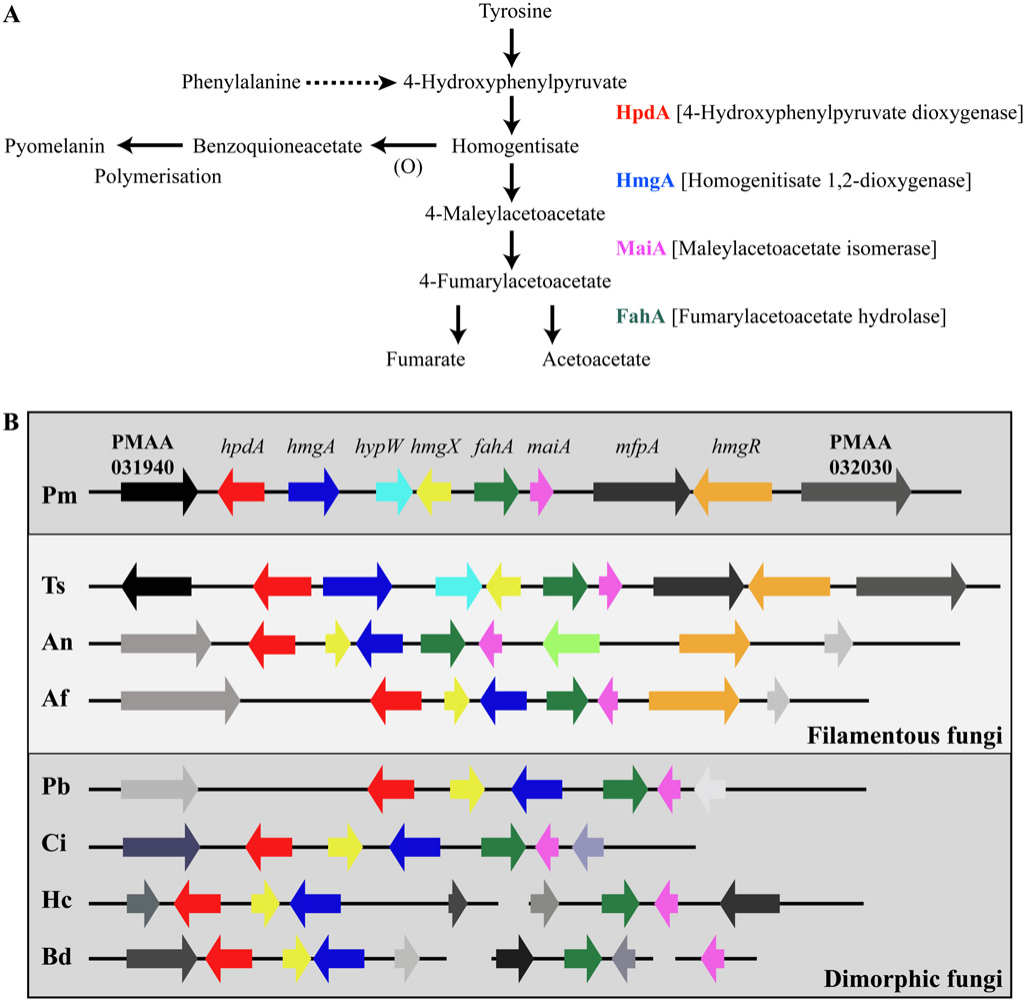
The catabolism of tyrosine. A. Tyrosine is catabolised to the toxic compound 4-hydroxyphenylpyruvate by an unknown mechanism. Phenylalanine can be degraded by the prephenate pathway to 4-hydroxyphenylpyruvate. 4-hydroxyphenylpyruvate dioxygenase (HpdA) catalyses the conversion of 4-hydroxyphenylpyruvate to homogentisate. Homogentisate can either be oxidized and polymerized to form the brown pigment pyomelanin or metabolised to 4-maleylacetoacetate by homogentisate 1,2-dioxygenase (HmgA). 4-maleylacetoacetate is metabolised to 4-fumarylacetoacetate by maleylacetoacetate isomerase (MaiA). Fumarlacetoacetate hydrolase (FahA) catalyses the conversion of 4-fumarylacetoacetate to acetoacetate and fumarate, which can be utilized as carbon sources via the TCA cycle. B. Genes required for the catabolism of tyrosine are located in a gene cluster which is conserved in filamentous (*Talaromyces stipitatus*; Ts, *Aspergillus nidulans*; An and *Aspergillus fumigatus*; Af, light grey box) and dimorphic (*P*. *marneffei*; Pm, *Paracoccidioides brasiliensis*; Pb, *Coccidioides immitis*; Ci, *Histoplasma capsulatum*; Hc and *Blastomyces dermatitidis*; Bd, dark grey boxes) fungi. Tyrosine catabolism genes are coloured as follows: *hpdA*; red, *hmgA*; blue, *hypW*; aqua, *hmgX*; yellow, *fahA*; green, *maiA*; pink and *hmgR*; orange. Flanking genes (PMAA_031940, PMAA_032030, TSTA_065640, TSTA_065560, AN1900, AN1892, AFUA_2G04190, AFUA_2G04270, PADG_08469, PADG_08463, CIMG_01309, CIMG_01315, HCEG_08529, HCEG_08533, HCEG_03254, HCEG_03257, BDDG_05746, BDDG_05738, BDDG_08624 and BDDG_12986) with no characterized role in tyrosine catabolism are shown in grey (same shade if orthologous). *hypW* is present only in *P*. *marneffei* (PMAA_032010) and *T*. *stipitatus* (TSTA_065580). *A*. *nidulans* contains an internal gene (AN1894) with no characterized role in tyrosine catabolism (light green). *T*. *stipitatus fahA* and *maiA* are misannotated in the *Talaromyces stipitatus* ATCC 10500 genomic database as a single fused gene named *fahA* (TSTA_065590). The cluster has been divided into two in *H*. *capsulatum* and *B*. *dermatitidis*. *P*. *brasiliensis*, *C*. *immitis*, *H*. *capsulatum* and *B*. *dermatitidis* lack a *hmgR* orthologue. Gene orthologues used to generate this Figure are as follows: *hpdA* (PMAA_031950, TSTA_065630, AN1899, AFUA_2G04200, PADG_08468, CIMG_01310, HCEG_08530 and BDDG_05744), *hmgA* (PMAA_031960, TSTA_065620, AN1897, AFUA_2G04220, PADG_08466, CIMG_01312, HCEG_08532 and BDDG_05741), *hypW* (PMAA_031970 and TSTA_065610), *hmgX* (PMAA_031980, TSTA_065600, AN1898, AFUA_2G04210, PADG_08467, CIMG_01311, HCEG_08531 and BDDG_05742), *fahA* (PMAA_031990, TSTA_065590, AN1896, AFUA_2G04230, PADG_08465, CIMG_01313, HCEG_03255 and BDDG_08623), *maiA* (PMAA_032000, AN1895, AFUA_2G04240, PADG_08464, CIMG_01314, HCEG_03256 and BDDG_08618), *mfpA* (PMAA_031010, TSTS_065580), and *hmgR* (PMAA_032020, TSTA_065570, AN1893 and AFUA_2G04262).

Tyrosine is assimilated via a conserved catabolic pathway that provides the fungus with both nitrogen, from a transamination reaction with α-ketoglutarate to produce glutamate, and carbon via production of fumarate and acetoacetate, which is used to generate acetyl-CoA that can then feed into the TCA cycle (Fig. 2A). Microarray-based expression profiling identified the *hpdA* gene as highly upregulated in yeast cells compared to hyphal cells [6]. Annotation of the 30Kb genomic region which encompasses *P*. *marneffei hpdA* shows the genes encoding additional enzymes required for the catabolism of tyrosine (*hmgA*, *maiA* and *fahA*) are located in a gene cluster (Fig. 2B). Tyrosine catabolism genes are also clustered in *Aspergillus* species [7, 8, 11]. The *P*. *marneffei* gene cluster also contains a gene (*hmgR*) encoding a Zn(II)2-Cys6 binuclear cluster transcription factor which has been shown to be required for expression of genes required for tyrosine catabolism in *A*. *fumigatus*, and a conserved gene, *hmgX*, which is postulated to act as an accessory factor to HpdA [5](Fig. 2B). The *P*. *marneffei* tyrosine cluster contains an additional ORF PMAA031970, we have named *hypW*, which is absent in the *Aspergilli* and other dimorphic pathogens. *hypW* is predicted to encode a hypothetical protein of unknown function which lacks any predicted domains (Fig. 2B). The *P*. *marneffei* tyrosine catabolism gene cluster also contains an additional gene, *mfpA* (PMAA_032010), encoding a putative alpha-1, 2-mannosidase family protein which is conserved in other fungi but located elsewhere in the genome (Fig. 2B). Blast searches of the *P*. *marneffei* genome also revealed the presence of paralogous genes scattered throughout the genome. A comparison of other fungal genomes revealed this is common to many species (Table 1). The *P*. *marneffei* genome contains a *hpdA* paralogue (PMAA_089170), four *hmgA* paralogues (PMAA_102000, PMAA_054730, PMAA_035450 and PMAA_080510) and two *fahA* paralogues (PMAA_080500 and PMAA_099050).

**Table 1:** Paralogues of tyrosine catabolic cluster genes.

Organism								
Homologue	Pm	Ts	An	Af	Hc	Pb	Bd	Ci
*hpdA*	2	2*	1	2	2	2	2	2
*hmgA*	5	4	4	2	1	1	1	1
*hypW*	1	1	0	0	0	0	0	0
*hmgX*	1	1	1	1	1	1	1	1
*fahA*	3	3	3	2	1^#^	1	1^#^	1
*maiA*	1	3^*a*^	1	1	1^#^	1	1^#^	1
*hmgR*	1	1	1	1	0	0	0	0

* The *hpdB* copy is truncated and lacks the cd07250 and cd08342 domains so is likely to be non-functional.

^#^ Copies not present in cluster.

^*a*^ *T*. *stipitatus fahA* and *maiA* are misannotated in the *Talaromyces stipitatus* ATCC 10500 genomic database as a single fused gene named *fahA* (TSTA_065590).

Organisms are as follows: Pm; *Penicillium marneffei*, Ts; *Talaromyces stipitatus*, An; *Aspergillus nidulans*; Af; *Aspergillus fumigatus*, Hc; *Histoplasma capsulatum* and Pb; *Paracoccidioides brasiliensis*.

To investigate if this cluster is conserved in other fungi, approximately 30Kb of the genomic region which encompasses the orthologue of *hpdA*, from *P*. *marneffei*’s closest sexual relative *T*. *stipitatus*, from the dimorphic fungal pathogens *P*. *brasiliensis*, *Coccidioides immitis*, *H*. *capsulatum* and *Blastomyces dermatitidis*, and the filamentous fungi *A*. *nidulans* and *A*. *fumigatus* was compared to that of *P*. *marneffei* (Fig. 2B). The homologues of *hmgA*, *maiA*, *fahA* and *hmgX* were within close proximity of *hpdA* in all species except *H*. *capsulatum* and *B*. *dermatitidis* in which the catabolic cluster was split into two and three, respectively, different genomic locations (Fig. 2B). *T*. *stipitatus fahA* and *maiA* are misannotated in the database as a single fused gene named *fahA* (TSTA_065590). The cluster in *T*. *stipitatus* also contains a homologue of *hypW* and *mfpA* (Fig. 2B). *P*. *brasiliensis*, *C*. *immitis*, *H*. *capsulatum* and *B*. *dermatitidis* also lacked a homologue of the Zn(II)2-Cys6 binuclear cluster transcription factor encoded by *hmgR*.

### The expression of tyrosine catabolic cluster genes is regulated by nitrogen source via the HmgR and AreA transcription factors

**Fig 3:**
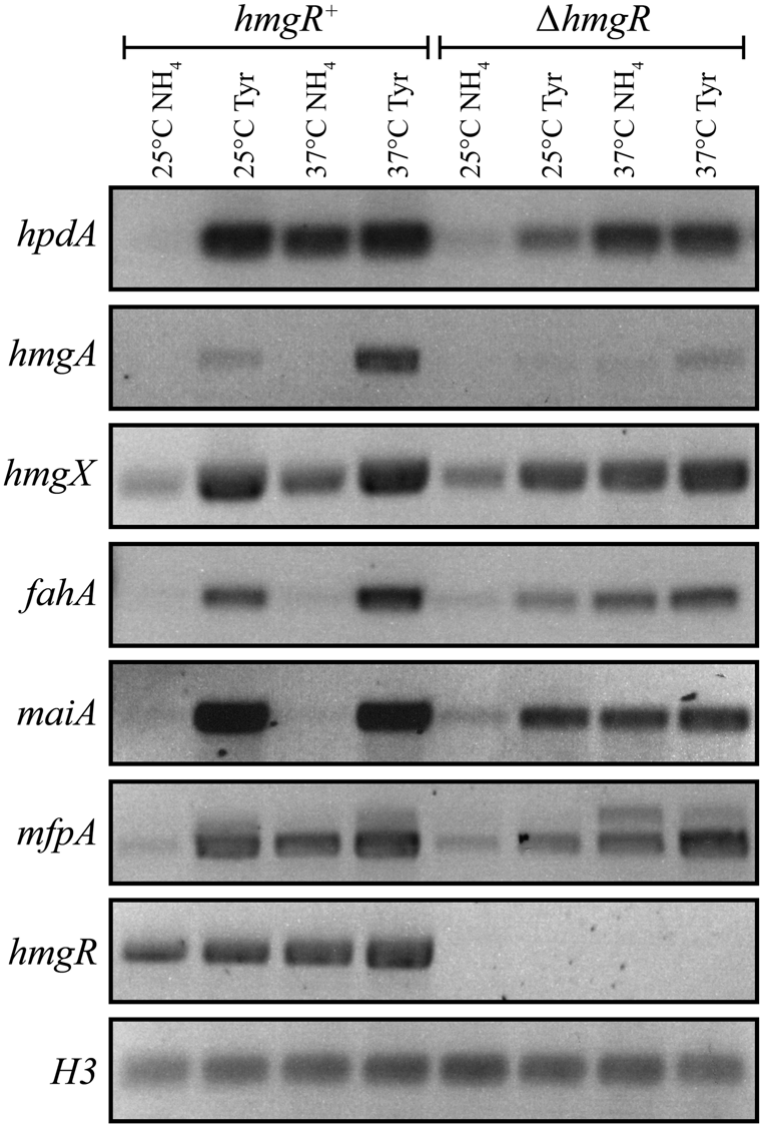
The tyrosine induced expression of genes of the catabolism cluster requires the HmgR transcription factor. RNA was isolated from wildtype (*hmgR* ^+^) and Δ*hmgR* strains grown in liquid culture for 2 days at 25°C or 6 days at 37°C and transferred into media containing ammonium (NH_4_) or tyrosine (Tyr) as the sole nitrogen source at 25°C or 37°C for 4 hours. Expression of *hpdA* (PMAA_031950), *hmgA* (PMAA_031960), *hmgX* (PMAA_031980), *fahA* (PMAA_031990), *maiA* (PMAA_032000), *mfpA* (PMAA_031010) and *hmgR* (PMAA_032020) was detected by RT PCR.

To investigate the expression of genes in the tyrosine catabolic cluster, RNA was isolated from cells grown in liquid culture for 2 days at 25°C (hyphal cells) or 6 days at 37°C (yeast cells) then transferred into medium containing ammonium or tyrosine as the sole nitrogen source at 25°C or 37°C for 4 hours. At both 25°C and 37°C, expression of *hmgA*, *hmgX*, *fahA* and *maiA* was low in the presence of ammonium and high in tyrosine suggesting that expression is induced in the presence of tyrosine (Fig. 3). Although *hpdA* showed the same pattern of expression at 25°C, at 37°C expression in ammonium and tyrosine was almost equivalent suggesting that unlike the other genes in the cluster, *hpdA* expression at 37°C is not repressed in the presence of a preferred nitrogen source (Fig. 3). The *hmgR* gene was expressed in the presence of both ammonium and tyrosine at both 25°C and 37°C, with expression only slightly higher in tyrosine (Fig. 3). Interestingly, expression of *mfpA*, the gene within the tyrosine metabolic cluster only in *P*. *marneffei* and *T*. *stipitatus* which is not predicted to have a role in tyrosine catabolism, was induced on tyrosine at both 25°C and 37°C (Fig. 3). This suggests that the cluster may also be under a more global level of regulation such as that mediated by chromatin effects and any gene captured within this genomic region may consequently come under it’s regulation. The analysis of *hypW* expression using RT-PCR primers spanning the predicted intron showed an expression pattern similar to the other genes of the tyrosine catabolic cluster (S3A Fig). However, the size of the RT PCR product suggested the predicted intron is not spliced. Examination of RNAseq data for this region confirmed the lack of this predicted intron and indicated that the predicted start site is incorrect (H. Weerasinghe and A. Andrianopoulos, personal communication)(S3B Fig). The small number of the RNA seq reads were also restricted to the 5’ region which is in close proximity to *hmgX* (163bp) (S3B Fig). Overall, this suggests that this ORF is not expressed under the conditions tested and the expression observed by RT PCR is due to read through from *hmgX*. Expression of the paralogues of tyrosine catabolic genes could not be detected under these conditions.

**Fig 4:**
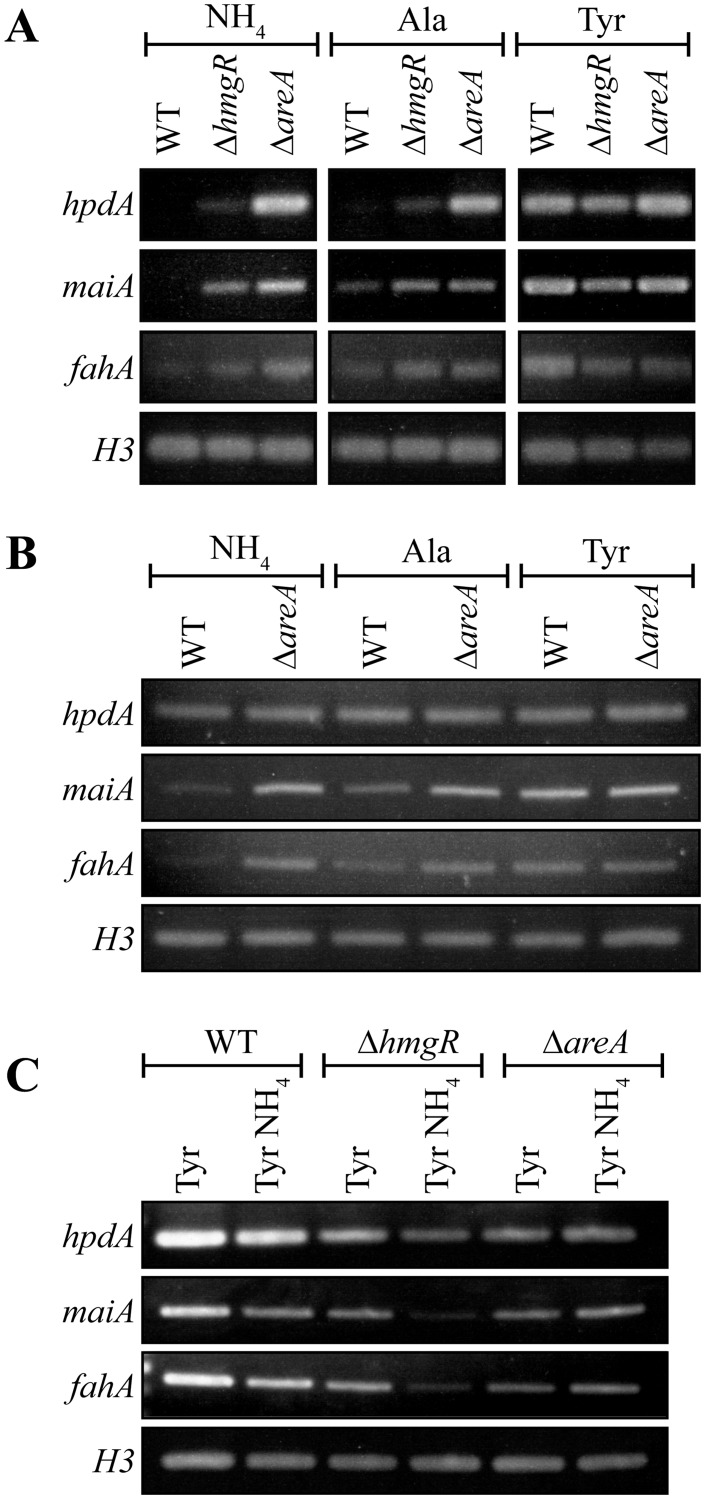
AreA negatively regulates expression of tyrosine catabolism genes. A. RNA was isolated from wildtype (WT), Δ*hmgR* and Δ*areA* strains grown in liquid culture for 2 days at 25°C (A and C) or 6 days at 37°C (B) and transferred into media containing ammonium (NH_4_), alanine (Ala) or tyrosine (Tyr) as the sole nitrogen source at 25°C (A) or at 37°C (B) for 4 hours, or media containing tyrosine (Tyr) or both tyrosine and ammonium (Tyr NH_4_) at 25°C for 4 hours (C). Expression of *hpdA*, *maiA*, *fahA* and a *H3* loading control was detected by RT PCR.

To investigate if the tyrosine-induced expression of the genes in the cluster is a result of a general response to limiting nitrogen levels or a specific induction by tyrosine, *hpdA*, *maiA* and *fahA* expression was also assessed in alanine at 25°C and 37°C. Expression of these genes was higher in alanine compared to ammonium but lower than tyrosine at both temperatures. This suggests that the increased expression observed in tyrosine is not solely due to derepression of tyrosine catabolic genes in response to general limiting nitrogen levels but rather specific induction due to the presence of tyrosine (Fig. 4A and B).

A gene encoding a Zn(II)2-Cys6 binuclear transcription factor, *hmgR*, is conserved in the tyrosine metabolic cluster across many fungi. This gene has been shown to be essential for tyrosine-induced expression of *hppD (hpdA* orthologue), *hmgA*, *hmgX*, *fahA* and *maiA* in *A*. *fumigatus* [5]. To investigate the role of *hmgR* in *P*. *marneffei*, the orthologous gene (PMAA_032020) was cloned and a deletion strain (Δ*hmgR*::*pyrG* ^*+*^ (G825)) was generated by transforming *P*. *marneffei* strain G816 (Δ*ligD niaD1 pyrG1*). To generate a complemented strain (Δ*hmgR hmgR* ^*+*^ (G867)), a Δ*hmgR pyrG* ^-^ (G864) strain was transformed with a 5.1 kb *hmgR* fragment targeted to the *P*. *marneffei pyrG* locus.

In contrast to *A*. *fumigatus*, deletion of *hmgR* in *P*. *marneffei* resulted in only the partial loss of induction of *hpdA*, *hmgA*, *hmgX*, *fahA* and *maiA* in medium with tyrosine as the sole nitrogen source at both 25°C and 37°C (Fig. 3). This suggests that this transcription factor is required, but not essential, for tyrosine-induced expression in *P*. *marneffei*. Interestingly, deletion of *hmgR* resulted in increased expression of *hpdA* at 25°C and *hmgA*, *hmgX*, *fahA* and *maiA* at both 25°C and 37°C in medium containing ammonium as the sole nitrogen source (Fig. 3). Therefore, in addition to inducing the expression of tyrosine catabolism genes in the presence of tyrosine, this transcription factor is also required to repress their expression in the presence of a preferred nitrogen source.

Nitrogen metabolite repression is a regulatory mechanism utilized by microbes to allow the preferential use of readily assimilated (preferred) nitrogen sources. The *areA* gene encodes a positively-acting GATA-type transcription factor which regulates the global response to limiting nitrogen conditions in *P*. *marneffei* and other fungi [29, 30]. To investigate the contribution of AreA regulation to the tyrosine catabolic cluster, the expression of *hpdA*, *maiA* and *fahA* was examined in wildtype, Δ*hmgR* and Δ*areA* at 25°C and 37°C. RNA was isolated from cells grown in liquid culture for 2 days at 25°C or 6 days at 37°C and transferred into medium containing ammonium, alanine or tyrosine as the sole nitrogen source at 25°C or 37°C for 4 hours. In wildtype, expression of *maiA* and *fahA* was barely detectable in ammonium, increased to a low level in alanine and strongly induced in tyrosine at both 25°C and 37°C (Fig. 4A and B). Expression levels of *hpdA* were similarly dependent on the nitrogen source at 25°C but constitutive at 37°C (Fig. 4A and B). Compared to wildtype, the expression of *hpdA*, *maiA* and *fahA* in the Δ*hmgR* mutant at 25°C was partially derepressed in ammonium and alanine and full induction in tyrosine was not observed (Fig. 4A). Surprisingly unlike wildtype and the Δ*hmgR* mutant, the expression of *hpdA*, *maiA* and *fahA* on either ammonium, alanine or tyrosine was equivalent in the Δ*areA* mutant at 25°C (Fig. 4A). This was unexpected given that AreA usually acts as a positive regulator of gene expression under conditions of limiting nitrogen. Likewise at 37°C, the expression of *maiA* and *fahA* was equivalent on either ammonium, alanine or tyrosine in the Δ*areA* mutant (Fig. 4B). Deletion of *areA* did not affect the highly constitutive expression of *hpdA* at 37°C (Fig. 4B). These results suggest that AreA is not acting to positively regulate expression of the tyrosine catabolic cluster in the presence of tyrosine. In addition, the increased expression of these genes in the Δ*areA* mutant on ammonium and alanine suggests that AreA is negatively regulating expression of the cluster in the absence of tyrosine which is contradictory to the current model of AreA function.

To investigate if genes of the tyrosine catabolic cluster are under nitrogen metabolite repression mediated by AreA, the expression of *hpdA*, *maiA* and *fahA* was assessed on tyrosine and both tyrosine and ammonium at 25°C in both wildtype, Δ*hmgR* and Δ*areA* (Fig. 4C). In wildtype, the levels of *hpdA*, *maiA* and *fahA* are lower on tyrosine and ammonium medium compared to tyrosine alone suggesting that these genes are under nitrogen metabolite repression (Fig. 4C). Although the overall level of induction is reduced in the Δ*hmgR* mutant, the levels of *hpdA*, *maiA* and *fahA* are lower on tyrosine and ammonium medium compared to tyrosine alone suggesting that HmgR is not playing a role in nitrogen metabolite repression (Fig. 4C). In contrast, in the Δ*areA* mutant the levels of *hpdA*, *maiA* and *fahA* expression are equivalent between tyrosine and ammonium medium compared to tyrosine alone indicating that *areA* is required during nitrogen metabolite repression to repress expression of the cluster in the presence of ammonium (Fig. 4C).

### Genes of the tyrosine catabolic cluster are required for hyphal growth on tyrosine as a nitrogen or carbon source at 25°C

**Fig 5:**
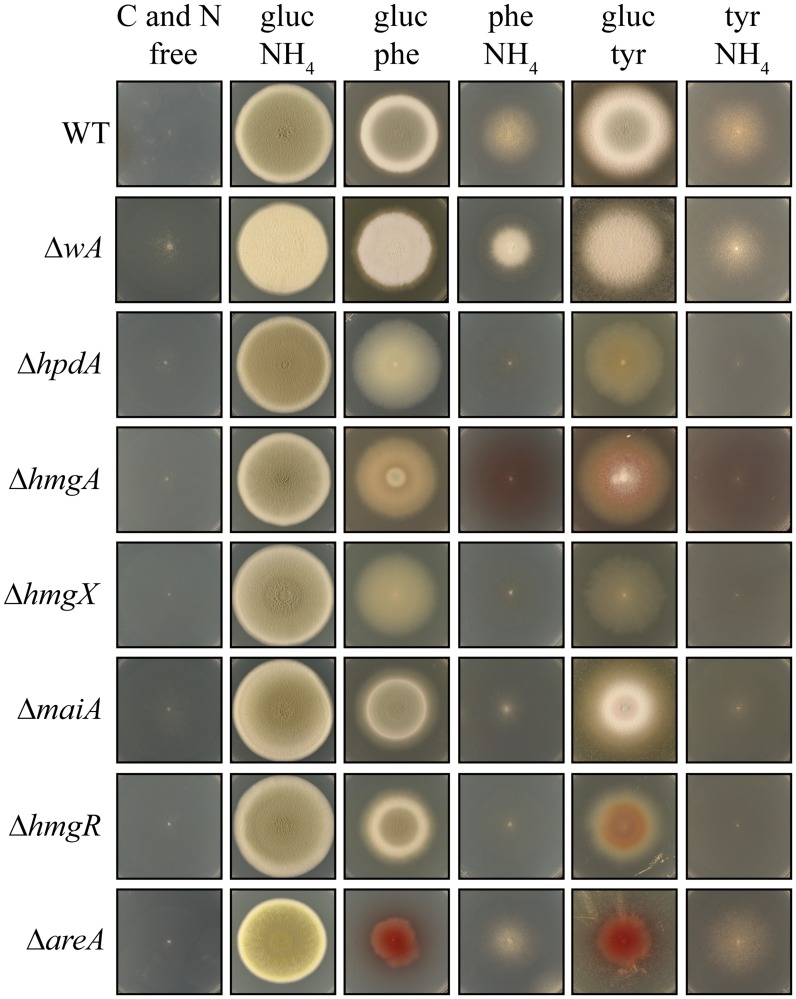
Genes of the tyrosine catabolic cluster are required for hyphal growth on tyrosine and phenylalanine as a nitrogen or carbon source at 25°C. Growth of the wildtype, Δ*wA*, Δ*hpdA*, Δ*hmgA*, Δ*hmgX*, Δ*maiA*, Δ*hmgR*, and Δ*areA* strains on carbon and nitrogen free medium (C and N free), on ammonium as the sole nitrogen source (gluc NH_4_), on phenylalanine as the sole nitrogen source (gluc phe), on phenylalanine as the sole carbon source (phe NH_4_), on tyrosine as the sole nitrogen source (gluc tyr) or on tyrosine as the sole carbon source (tyr NH_4_) after 14 days at 25°C.

To determine the role of genes present in the tyrosine catabolic cluster, *hpdA*, *hmgA*, *hypW*, *hmgX*, *maiA* and *mpfA* were cloned and deleted. Due to the fact that an intermediate in the tyrosine catabolism pathway, homogentisate, can be oxidized and polymerized to form the brown pigment pyomelanin, the *wA* gene required for the synthesis of DHN melanin was also cloned and deleted for comparison. As expected, the Δ*wA* strain appears white due to the absence of DHN melanin in asexual spores (conidia) (Fig. 5). To confirm the mutant phenotype was a result of the gene deletion events, deletions strains were complemented with the wildtype gene targeted to either *pyrG* or *niaD*.

Tyrosine can be utilized as both nitrogen and carbon sources for growth. In *A*. *fumigatus*, an L-amino oxidase is thought to catalyse the conversion of tyrosine to the α-keto acid, liberating ammonium as a nitrogen source. However, the *P*. *marneffei* genome lacks genes encoding L-amino oxidases so it is likely that a transamination reaction of tyrosine with α-ketoglutarate to produce glutamate as a nitrogen source is the first catabolic step. The enzyme that performs this reaction is currently unknown. The catabolism of tyrosine also produces fumarate and acetoacetate, which is further catabolised to acetyl-CoA, which feed into the TCA cycle to provide carbon (Fig. 2A). Phenylalanine is also catabolised via the tyrosine catabolism pathway to provide both nitrogen and carbon, although the enzymatic steps required to convert it to 4-hydroxyphenylpyruvate remain unclear. Growth of the wildtype, Δ*wA*, Δ*hpdA*, Δ*hmgA*, Δ*hmgX* and Δ*maiA* strains was assessed at 25°C on a range of media in which the sole carbon source was either glucose, tyrosine or phenylalanine and the sole nitrogen source was either ammonium, tyrosine or phenylalanine or on medium lacking both a nitrogen and carbon source. Wildtype and the Δ*wA* strain grew well on tyrosine or phenylalanine as a sole nitrogen or carbon source (Fig. 5). Compared to wildtype and the Δ*wA* strain, the Δ*hpdA*, Δ*hmgA*, Δ*hmgX* and Δ*maiA* strains showed reduced growth on tyrosine and phenylalanine as the sole nitrogen source which suggests a feedback loop regulates the nitrogen-liberating first step in the catabolism of tyrosine (Fig. 5). Unlike wildtype and the Δ*wA* strain, the Δ*hpdA*, Δ*hmgA*, Δ*hmgX* and Δ*maiA* strains showed no growth on tyrosine or phenylalanine as the sole carbon source (Fig. 5). The medium became pigmented when the Δ*hmgA* mutant was grown on tyrosine or phenylalanine as the sole nitrogen or carbon source (Fig. 5). This suggests that accumulated homogentisate is being oxidized to produce pyomelanin, as has been observed in the *Aspergilli* [10, 11, 31]. Reintroduction of the wildtype gene to generate the Δ*hmgA hmgA* ^*+*^, Δ*hmgX hmgX* ^*+*^, Δ*maiA maiA* ^*+*^ complemented strains restored growth on tyrosine and phenylalanine as the sole nitrogen and carbon source at 25°C but to a level slightly below that of wildtype (S1A Fig). The Δ*hpdA hpdA* ^*+*^ strain did not show the same extent of restoration of growth on tyrosine and phenylalanine as the sole carbon source, indicating either the complementation construct may lack some regulatory sequences required for full expression or the location of the gene in the cluster, as opposed to an ectopic site, is important for it’s regulation (S1A Fig). The growth phenotypes of the Δ*hpdA* and Δ*hmgA* strains suggests that there is no functional overlap between these genes and the paralogues located elsewhere in the genome with respect to this catabolic pathway. *To confirm that these paralogues do not have a role in tyrosine catabolism*, *hpdB was cloned and deleted*. This gene was selected as *hpdA* has only a single paralogue in the genome, whereas, *hmgA* has four paralogues. Unlike Δ*hpdA*, the Δ*hpdB* strain was indistinguishable from wildtype on tyrosine or phenylalanine as either the sole nitrogen or carbon source at 25°C and 37°C supporting the hypothesis that there is no functional overlap between *hpdA* and *hpdB* (S1B Fig and S4B Fig).

Accumulation of intermediates of tyrosine catabolism results in cellular toxicity due to the production of intermediary metabolites or their spontaneous degradation products (4-hydroxyphenlpyruvic acid in *hpdA* mutants, homogentistic acid in *hmgA* mutants and succinylacetone and succinylacetoacetate from fumarylacetoacetate in *fahA* mutants) [7, 8, 10, 32, 33]. To investigate if toxic metabolites are accumulating in the *P*. *marneffei* tyrosine catabolism mutants, the wildtype, Δ*hpdA*, Δ*hmgA*, Δ*hmgX* and Δ*maiA* strains were grown on tyrosine medium also containing the non-repressive carbon sources sorbitol, lactose, acetate or proline. In contrast to wildtype, the Δ*hpdA* and Δ*hmgA* strains showed no growth under these conditions indicating that toxic intermediates are accumulating in these strains (S2 Fig). Growth of the Δ*hmgX* strain was less than wildtype but greater than the Δ*hpdA* and Δ*hmgA* mutants. This suggests that the levels of toxic intermediates are lower in this strain compared to Δ*hpdA* and Δ*hmgA* (S2 Fig). Growth of the Δ*maiA* was unaffected on tyrosine medium also containing the non-repressive carbon sources suggesting that toxic intermediates are not accumulating in this strain (S2 Fig). These results are in contrast to *A*. *nidulans* in which deletion of *hpdA* and *maiA*, but not *hmgA*, results in cellular toxicity [10, 32, 33]. These results suggest putative differences between the metabolites derived from homogentisate and 4-malelyacetate in *P*. *marneffei* versus *A*. *nidulans*.

To assess if the genes within the cluster that are not conserved in other species also have a role in tyrosine catabolism in *P*. *marneffei*, growth of the Δ*mfpA* and Δ*hypW* strains was assessed at 25°C on glucose, tyrosine or phenylalanine as the sole carbon source, ammonium, tyrosine or phenylalanine as the sole nitrogen source or on medium lacking both a nitrogen and carbon source. As expected, the Δ*mfpA* strain was indistinguishable from wildtype under these conditions indicating that despite being transcriptionally regulated in response to the presence of tyrosine *mfpA* is not required for tyrosine catabolism (S1B Fig). However, unexpectedly, the Δ*hypW* strain showed reduced growth on tyrosine and phenylalanine as the sole nitrogen or carbon source compared to wildtype (S3C Fig). Due to the low transcriptional activity in this region and the ORF’s close proximity to *hmgX*, we hypothesized that deletion of this ORF may be affecting *hmgX* expression. To assess this, *hmgX* expression was evaluated in the Δ*hypW* strain at 25°C in ammonium and tyrosine as the sole nitrogen source. Deletion of *hypW* reduced expression of *hmgX* under both conditions suggesting that the reduced growth on tyrosine as the sole nitrogen source is a consequence of reduced *hmgX* expression rather than indicating a role for *hypW* in tyrosine catabolism (S3D Fig). In support of this hypothesis, the introduction of a construct containing both the *hypW* and *hmgX* wildtype genes complemented the phenotype of the Δ*hypW* strain at both 25°C and 37°C, whereas, introduction of a construct containing wildtype *hypW* and a truncated *hypX* lacking the start codon, did not complement this growth phenotype (S3C Fig). Therefore, *hypW* is not required for tyrosine catabolism

To further investigate the role of regulatory genes on the tyrosine catabolic cluster, the growth of the Δ*hmgR* and Δ*areA* strains was assessed at 25°C on glucose, tyrosine or phenylalanine as the sole carbon source, ammonium, tyrosine or phenylalanine as the sole nitrogen source or on medium lacking both a nitrogen and carbon source. Compared to wildtype, the Δ*hmgR* strain showed reduced growth on tyrosine and phenylalanine as the sole nitrogen or carbon source (Fig 5). Reintroduction of the wildtype gene in the Δ*hmgR hmgR* ^*+*^ complemented strain restored growth on tyrosine and phenylalanine as the sole nitrogen and carbon source at 25°C (S2A Fig). This growth reduction correlates with the reduced expression of tyrosine catabolic genes on tyrosine as a sole nitrogen source in the Δ*hmgR* mutant (Fig. 3). To assess if the growth reduction of the Δ*hmgR* strain was specific to tyrosine and phenylalanine, the Δ*hmgR* strain was also grown on a variety of amino acids as the sole nitrogen source at 25°C and 37°C. No reduction in growth was observed on any other amino acids tested. The Δ*areA* strain showed reduced growth on tyrosine or phenylalanine as the sole nitrogen source but was unaffected on tyrosine and phenylalanine as a carbon source (Fig. 5). As AreA does not directly regulate expression of the tyrosine catabolic genes in the gene cluster (Fig. 4), this result suggests that the deamination step is AreA dependent and in it’s absence there can be no metabolites to flux through the pathway.

### Yeast growth at 37°C on tyrosine as a nitrogen or carbon source requires genes of the tyrosine catabolic cluster

**Fig 6:**
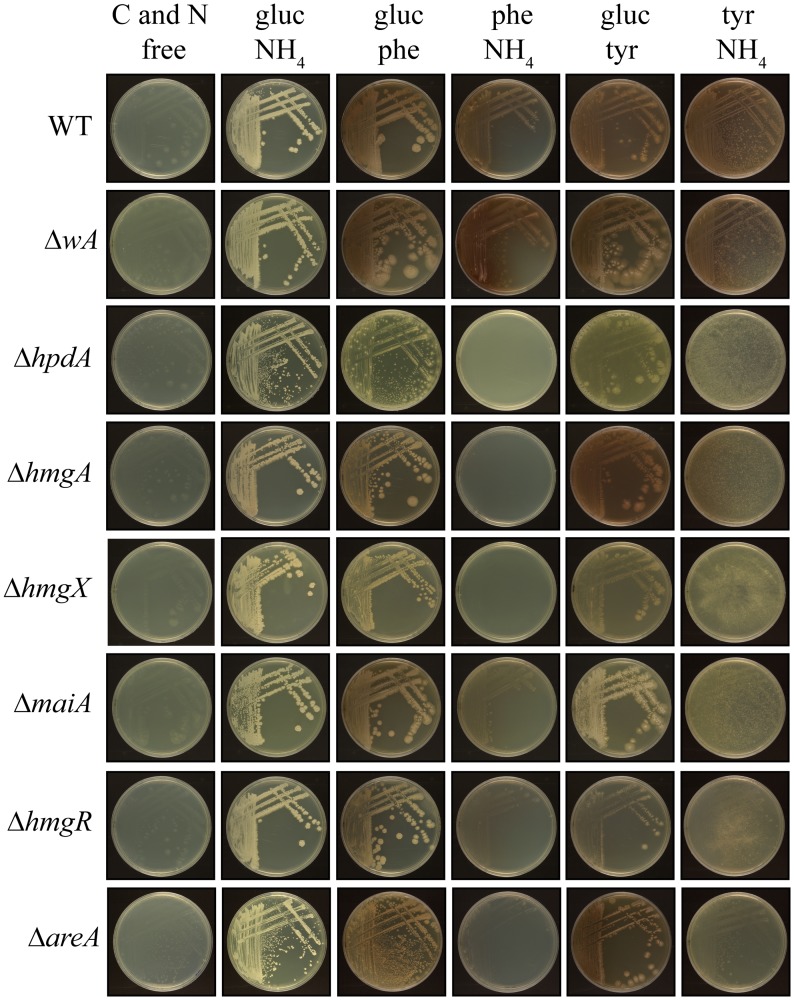
Growth of yeast cells on tyrosine and phenylalanine as a nitrogen or carbon source at 37°C requires genes of the tyrosine catabolic cluster. Growth after 14 days at 37°C of the wildtype, Δ*wA*, Δ*hpdA*, Δ*hmgA*, Δ*hmgX*, Δ*maiA*, Δ*hmgR* and Δ*areA* strains on carbon and nitrogen free medium (C and N free), on ammonium as the sole nitrogen source (gluc NH_4_), on phenylalanine as the sole nitrogen source (gluc phe), on phenylalanine as the sole carbon source (phe NH_4_), on tyrosine as the sole nitrogen source (gluc tyr) or on tyrosine as the sole carbon source (tyr NH_4_).

To assess if the tyrosine catabolic gene cluster is required for yeast-phase growth on tyrosine at 37°C, the wildtype, Δ*wA*, Δ*hpdA*, Δ*hmgA*, Δ*hmgX* and Δ*maiA* strains were grown on glucose, tyrosine or phenylalanine as the sole carbon source and ammonium, tyrosine or phenylalanine as the sole nitrogen source at 37°C. Growth of wildtype on tyrosine or phenylalanine as a sole nitrogen or carbon source at 37°C is relatively robust when compared to growth on the preferred nitrogen and carbon sources of ammonium and glucose, respectively (Fig. 6). Compared to wildtype and the Δ*wA*, the Δ*hpdA* and Δ*hmgX* strains showed reduced growth on tyrosine and phenylalanine as the sole nitrogen source at 37°C (Fig. 6). However unlike 25°C, the Δ*hmgA* and Δ*maiA* strains showed no growth reduction on tyrosine and phenylalanine as the sole nitrogen source at 37°C. This difference suggests that the feedback regulation of the pathway observed at 25°C is not operating at 37°C, possibly because of a higher demand for intermediate metabolites such as homogentisate for pyomelanin production (Fig. 6). In contrast to wildtype and the Δ*wA*, the Δ*hpdA*, Δ*hmgA*, Δ*hmgX* and Δ*maiA* strains showed no growth on tyrosine or phenylalanine as the sole carbon source (Fig. 6). In the absence of growth on tyrosine as a carbon source, the un-utilized tyrosine in the medium crystalizes (Fig. 6). The Δ*hmgA hmgA* ^*+*^, Δ*hmgX hmgX* ^*+*^, Δ*maiA maiA* ^*+*^ complemented strains displayed growth almost to the same extent as wildtype on tyrosine and phenylalanine as the sole nitrogen and carbon source at 37°C (S4A Fig). Like at 25°C, the Δ*hpdA hpdA* ^*+*^ strain did not show a complete restoration of wildtype growth supporting the hypothesis that the position of the gene in the cluster is important for it’s regulation (S4A Fig).

To assess if *mfpA* has a role in tyrosine catabolism in *P*. *marneffei* at 37°C, growth of the Δ*mfpA* strain was assessed on glucose, tyrosine or phenylalanine as the sole carbon source and ammonium, tyrosine or phenylalanine as the sole nitrogen source. As expected, the Δ*mfpA* strain was indistinguishable from wildtype under these conditions indicating that it is not required for tyrosine catabolism (S4B Fig).

To investigate the role of regulatory genes on the tyrosine catabolic cluster at 37°C, the growth of the Δ*hmgR* and Δ*areA* strains was assessed at 37°C on glucose, tyrosine or phenylalanine as the sole carbon source and ammonium, tyrosine or phenylalanine as the sole nitrogen source. Compared to wildype, the Δ*hmgR* strain showed reduced growth on tyrosine and phenylalanine as the sole nitrogen or carbon source at 37°C and reintroduction of the wildtype gene restored growth (Fig. 6 and S4 Fig). This result is consistent with the reduced expression of tyrosine catabolic genes on tyrosine as a sole nitrogen source in the Δ*hmgR* mutant at 37°C (Fig. 3). The Δ*areA* strain showed reduced growth on tyrosine or phenylalanine as both the sole nitrogen and carbon source (Fig. 6).

### Pyomelanin production requires genes of the tyrosine catabolic cluster

**Fig 7:**
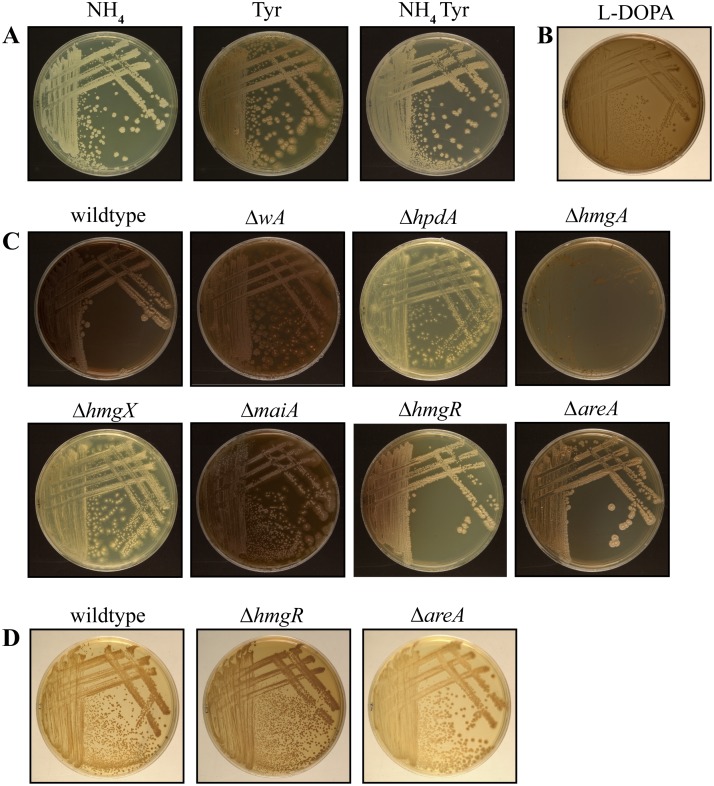
Pyomelanin produced via the tyrosine catabolism pathway is under nitrogen metabolite repression. A. Wildtype grown for 14 days at 37°C on ANM plus ammonium (NH_4_) or tyrosine (Tyr) as the sole nitrogen source or plus both ammonium and tyrosine (NH_4_ Tyr). Pyomelanin production via tyrosine catabolism is under nitrogen metabolite repression. B. Wildtype P. marneffei grown on L-DOPA medium for 14 days at 37°C. C. Pyomelanin formation on ANM plus alanine and tyrosine for 14 days at 37°C in the wildtype, Δ*wA*, Δ*hpdA*, Δ*hmgA*, Δ*hmgX*, Δ*maiA*, Δ*hmgR and* Δ*areA* strains after 14 days growth at 37°C. D. Wildtype, Δ*hmgR* and Δ*areA* grown for 14 days at 37°C on ANM plus ammonium and tyrosine. The Δ*hmgR* mutant produces increased pyomelanin and the Δ*areA* mutant produces decreased pyomelanin under this growth condition.

Tyrosine is an important precursor in the formation of two different types of melanin; DOPA melanin via the metabolism of tyrosine to form DOPA and pyomelanin through the oxidation and polymerization of homogentisate during tyrosine catabolism [10, 11]. To assess if *P*. *marneffei* can produce melanin from tyrosine, the wildtype strain was grown on medium containing ammonium or tyrosine as the sole nitrogen source, medium containing both ammonium plus tyrosine or tyrosine plus alanine as nitrogen sources or on L-DOPA at 37°C. *P*. *marneffei* was unpigmented on medium containing ammonium as a sole nitrogen source (Fig. 7A). In contrast, a brown pigment was evident on medium containing tyrosine as the sole nitrogen source, suggesting that this may be a melanin produced from tyrosine (Fig. 7A). This pigment was confirmed to be melanin by testing resistance to chemical degradation by boiling in acid (Materials and Methods)[23] (S5A Fig). If both ammonium and tyrosine are present the amount of melanization is greatly reduced suggesting that some of the genes required for production of this melanin are under nitrogen metabolite repression (Fig. 7A). The amount of melanization on tyrosine increases with the addition of another non-preferred nitrogen source such as alanine (Fig. 7C). Wildtype *P*. *marneffei* also becomes pigmented on L-DOPA medium indicating that *P*. *marneffei* is capable of producing DOPA melanin (Fig. 7B). To assess if deletion of genes of the tyrosine catabolic cluster affects the production of DOPA melanin, the wildtype, deletion and complementation strains were grown at 37°C on L-DOPA. The production of DOPA melanin was indistinguishable amongst all of the strains except Δ*areA*, which showed a decrease in DOPA melanin production (S5C Fig).

To investigate if pyomelanin production contributes to the melanization observed on tyrosine medium, the wildtype, Δ*wA*, Δ*hpdA*, Δ*hmgA*, Δ*hmgX* and Δ*maiA* strains were examined after growth at 37°C on both tyrosine as a sole nitrogen source (Fig. 6) and tyrosine plus alanine as nitrogen sources (Fig. 7C). In contrast to wildtype and Δ*wA*, the Δ*hpdA* and Δ*hmgX* strains produced no visible melanin at 37°C when grown on tyrosine (Fig. 5 and 6C). This suggests that although capable of producing DOPA melanin from tyrosine, the major melanin produced by *P*. *marneffei* on medium containing tyrosine is the allomelanin pyomelanin produced during tyrosine catabolism. This was further confirmed by testing if melanin particles could be isolated from the Δ*hpdA* mutant grown on tyrosine as the sole nitrogen source for 14 days at 37°C (Materials and Methods). In contrast to wildtype under these growth conditions, no melanin particles were observed in the Δ*hpdA* mutant after boiling in acid (S5A Fig). This confirms that the melanin produced by *P*. *marneffei* on tyrosine is pyomelanin. The Δ*maiA* strain showed a mild reduction in pyomelanin production compared to the wildtype (Fig. 6 and 7C). The Δ*hmgA* strain showed increased pyomelanin production on tyrosine as the sole nitrogen source (Fig. 6). The Δ*hmgA* strain failed to grow on medium containing both tyrosine and alanine (Fig. 7C), despite being able to grow on alanine or tyrosine when provided as sole nitrogen sources (Fig. 6). This suggests that like at 25°C toxic metabolites are accumulating in the Δ*hmgA* mutant, when grown with an additional non-repressing carbon source such as alanine. The Δ*hpdA* and Δ*hmgX* strains also showed reduced growth compared to wildtype on this medium suggesting some cellular toxicity, however this was to a lesser extent than at 25°C (Figs. 7C and S2 Fig). This suggests that fewer toxic metabolites accumulate in these mutants at 37°C possibly as a result of an increased demand for pyomelanin production via homogentisate. Reintroduction of the wildtype gene in the Δ*hpdA hpdA* ^*+*^, Δ*hmgA hmgA* ^*+*^, Δ*hmgX hmgX* ^*+*^ and Δ*maiA maiA* ^*+*^ complemented strains restored pyomelanin production on tyrosine as a sole nitrogen source (S4A Fig).

To investigate if genes within the cluster which do not play a role in tyrosine catabolism and gene paralogues outside the cluster are required for pyomelanin production, the Δ*mfpA* and Δ*hpdB* strains were also grown on medium containing tyrosine as the sole nitrogen source and medium with both tyrosine and alanine as the nitrogen sources at 37°C. Consistent with no role in tyrosine catabolism, the Δ*mfpA* and Δ*hpdB* mutants were indistinguishable from wildtype under these conditions (S4B Fig).

Wildtype *P*. *marneffei* appears pigmented on the standard medium of brain heart infusion (BHI) from growth at 37°C. BHI is comprised of bovine brain and heart tissue that is postulated to be rich in phenolic compounds [23]. To assess if pyomelanin contributes to pigmentation on BHI, the wildtype, Δ*wA*, Δ*hpdA*, Δ*hmgA*, Δ*hmgX*, Δ*maiA* and complemented control strains were grown at 37°C on BHI for 5 days. The Δ*hpdA* and Δ*hmgX* strains showed a large reduction in melanisation on BHI medium, whereas, the Δ*hmgA* strains showed an increase in melanisation (S5B Fig). The Δ*maiA* strain displayed a minor decrease in melanisation and the complemented strains were indistinguishable from wildtype (S5 Fig). This suggests that pyomelanin contributes to some, but not all, of the melanisation observed when *P*. *marneffei* is grown on BHI medium. As expected, the Δ*wA* strain was also comparable with wildtype, suggesting that DHN melanin is not contributing to the melanisation observed on BHI medium (S5B Fig).

Deletion of *hmgR* results in reduced expression of the tyrosine catabolic cluster genes in the presence of tyrosine at both 25°C and 37°C (Figs. 3 and 4). To assess if this decreased expression translates into a visible decrease in pyomelanin production, the Δ*hmgR* and Δ*hmgR hmgR* ^*+*^ strains were grown on medium containing both alanine and tyrosine at 37°C. Compared to wildtype and the Δ*hmgR hmgR* ^*+*^ complemented strain, the Δ*hmgR* mutant showed decreased pyomelanin production suggesting that *hmgR* is required to positively regulate pyomelanin production in the presence of tyrosine (Fig. 7C). The Δ*hmgR* mutant also showed a reduction of melanization on BHI medium (S5 Fig). Deletion of *hmgR* also leads to partial derepression of tyrosine catabolic cluster genes in the presence of ammonium (Figs. 3 and 4A and C). To investigate if this derepression results in inappropriate pyomelanin production, the wildtype, Δ*hmgR* and Δ*hmgR hmgR* ^*+*^ strains were also grown on ammonium plus tyrosine medium at 37°C. Compared to wildtype and the Δ*hmgR hmgR* ^*+*^ complemented strain, the Δ*hmgR* mutant produced increased pyomelanin production on ammonium plus tyrosine medium at 37°C consistent with the partial derepression of gene expression (Fig. 7D).

Deletion of *areA* also leads to derepression of tyrosine catabolic cluster genes in the presence of ammonium at 25°C and 37°C and a complete loss of nitrogen metabolite repression in ammonium plus tyrosine medium at 25°C (Fig. 4A, B and C). To investigate if this derepression also results in inappropriate pyomelanin production in these strains, the wildtype and Δ*areA* strains were grown on ammonium plus tyrosine medium at 37°C. In contrast to the Δ*hmgR* mutant, no increase in pyomelanin production was observed in the Δ*areA* strain on ammonium plus tyrosine medium (Fig. 7D). Rather, the Δ*areA* strain produced less pyomelanin under this growth condition (Fig. 7D). The Δ*areA* strain also appeared slightly less melanised on BHI medium (S5 Fig). Similarly, deletion of *areA* lead to reduced pyomelanin production on alanine plus tyrosine medium (Fig. 7C). These results support the previous hypothesis that the deamination step is AreA dependent and in a Δ*areA* mutant there is reduced metabolic flux through the pathway.

### Pyomelanin protects *P*. *marneffei* against oxidative stress

**Fig 8:**
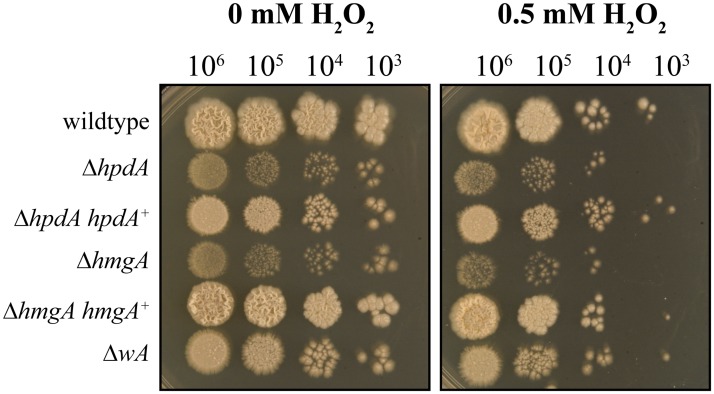
Pyomelanin is required for resistance to oxidative stress in *P*. *marneffei*. Serial dilutions of conidial suspensions of the wildtype, Δ*hpdA*, Δ*hpdA hpdA* ^+^, Δ*hmgA*, Δ*hmgA hmgA* ^+^ and Δ*wA* strains dropped onto medium containing tyrosine and 0 or 0.5 mM H_2_O_2_ and incubated for 6 days at 37°C.

Both DHN- and DOPA-melanin have been shown to protect fungal cells from reactive oxygen species (ROS) produced by host innate immune cells [12–15, 20]. To investigate if pyomelanin plays a similar role, the wildtype, Δ*wA*, Δ*hpdA*, Δ*hmgA*, and complemented control strains were grown for 6 days at 37°C on medium containing tyrosine as the nitrogen source and varying concentrations of hydrogen peroxide (H_2_O_2_) (Materials and Methods). The Δ*hpdA* and Δ*hmgA* strains showed increased sensitivity to H_2_O_2_ compared to the *wildtype*, Δ*hpdA hpdA* ^*+*^ and Δ*hmgA hmgA* ^*+*^ complemented strains (Fig. 8). The *wA* gene, which encodes the polyketide synthase required for DHN melanin production in conidia, is required for resistance to H_2_O_2_ in *A*. *fumigatus* [15, 20]. However, only a very mild sensitivity to H_2_O_2_ was observed in the *P*. *marneffei* Δ*wA* strain and this was much lower than the Δ*hpdA* and Δ*hmgA* strains (Fig. 8).

### Unlike the other genes of the tyrosine catabolic cluster *hpdA* is required for infectious growth in macrophages

**Fig 9:**
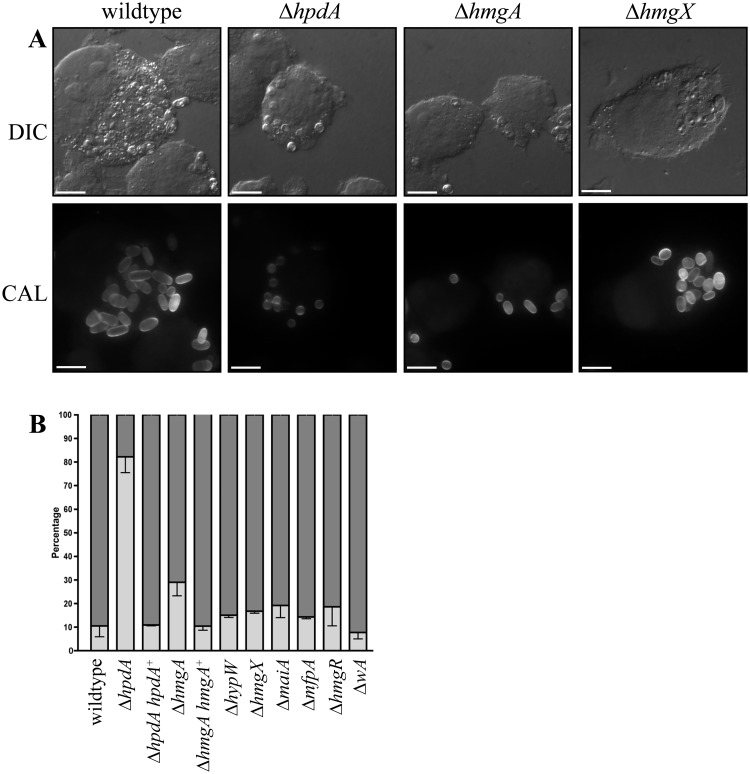
*ex vivo* growth in macrophages. Conidia of the wildtype, Δ*hpdA*, Δ*hpdA hpdA* ^*+*^, Δ*hmgA*, Δ*hmgA hmgA* ^*+*^, Δ*hypW*, Δ*hmgX*, Δ*maiA*, Δ*mfpA*, Δ*hmgR* and Δ*wA* strains were used to infect J774 murine macrophages and growth was assessed 24 hours post-infection. A. After 24 hours, macrophages infected with wildtype conidia contain numerous yeast cells dividing by fission. In contrast, ungerminated conidia were predominately observed in macrophages infected with conidia of the Δ*hpdA* mutant 24 hours post-infection. The Δ*hmgA* strain showed a small increase in the number of ungerminated conidia and a small decrease in the number of yeast cells. The Δ*hmgX* mutant was indistinguishable from wildtype. B. Quantitation of the percentage of ungerminated conidia and yeast cells in macrophages infected with wildtype, Δ*hpdA*, Δ*hpdA hpdA* ^*+*^, Δ*hmgA*, Δ*hmgA hmgA* ^+^, Δ*hypW*, Δ*hmgX*, Δ*maiA*, Δ*mfpA*, Δ*hmgR* and Δ*wA* 24 hours post-infection. Dark grey indicates the percentage of yeast cells, whereas, light grey represents the percentage of ungerminated conidia.

The addition of the HpdA inhibitor NTBC prevented *P*. *marneffei* yeast cell production during macrophage infection suggesting *hpdA* is required for *ex vivo* growth (Fig. 1). To confirm the required for *hpdA* during *ex vivo* growth and to assess if the other genes of the tyrosine catabolic cluster are also required for *ex vivo* growth, conidia of the wildtype, Δ*wA*, Δ*hpdA*, Δ*hmgA*, Δ*hypW*, Δ*hmgX*, Δ*maiA*, Δ*mfpA* and Δ*hmgR* strains were used to infect murine J774 macrophages and examined 24 hours post-infection. A control of wildtype conidia incubated in macrophage medium alone was also performed. After 24 hours, macrophages infected with wildtype conidia contain numerous yeast cells dividing by fission (Fig. 9A). In contrast, only 2.67 ± 0.70% of conidia had germinated in the macrophage medium control. This suggests that any growth of *P*. *marneffei* observed within macrophages is due to the derivation of nutrients from the host macrophage. In contrast to wildtype and consistent with the NTBC experiments, ungerminated conidia were predominately observed in macrophages infected with conidia of the Δ*hpdA* mutant 24 hours post-infection (Fig. 9A and B). In macrophages infected with the Δ*hpdA* strain, 82.2±6.78% of conidia remained ungerminated and only 17.4±6.57% germinated to produce yeast cells (compared to 10.6±4.65% ungerminated conidia and 89.5±4.65% yeast cells for wildtype) (Fig. 9B). This phenotype was complemented by reintroduction of *hpdA* ^+^ in the Δ*hpdA hpdA* ^*+*^ strain (10.9±0.41% ungerminated conidia and 88.4±0.66 yeast cells). No differences in germination rates were observed between the wildtype and Δ*hpdA* strain *in vitro*. Despite the Δ*hpdA* and Δ*hmgX* mutants having indistinguishable growth and pigmentation phenotypes on tyrosine, the Δ*hmgX* strain produced normal numbers of yeast cells *ex vivo* (Fig. 9A and B). The Δ*hmgA* strain showed a small increase in the number of ungerminated conidia (29.0±5.78%) and consequently a small decrease in the number of yeast cells (69.2±5.72%) produced at 24 hours post-infection (Fig. 9A and B). This phenotype was complemented by reintroduction of *hmgA* ^+^ in the Δ*hmgA hmgA* ^*+*^ strain (10.4±1.75% ungerminated conidia and 89.6±1.75% yeast). The production of yeast cells in macrophages infected with Δ*wA*, Δ*hypW*, Δ*hmgX*, Δ*maiA*, Δ*mfpA* and Δ*hmgR* conidia was indistinguishable from wildtype, suggesting that tyrosine and phenylalanine catabolism as a source of carbon is not required for the initial stages of infection (Fig. 9B). Interestingly, the Δ*wA* strain produced numerous yeast cells at levels equivalent to wildtype after 24 hours infection (85.2±4.97% compared to 89.5±4.65% for wildtype) despite a previous report that suggested decreased expression of this gene using RNAi results in a decrease in virulence in a mouse model of infection (Fig. 9B) [16].

To observe if Δ*hpdA* conidia germinate with a longer period of incubation, conidia of the wildtype, Δ*hpdA* and Δ*hpdA hpdA* ^+^ strains were used to infect murine J774 macrophages and examined 48 hours post-infection. After 48 hours, macrophages infected with wildtype and Δ*hpdA hpdA* ^+^ conidia contain prolific numbers of yeast cells dividing by fission (wildtype 1.43±0.98% ungerminated conidia, 98.6±0.98% yeast cells and Δ*hpdA hpdA* ^*+*^ 0.75±0.48 ungerminated conidia and 99.3±0.48% yeast cells). Conidia of the Δ*hpdA* strain have begun to germinate into yeast cells by 48 hours but show reduced cellular proliferation (38.5±5.71% ungerminated conidia, 61.3±5.63% yeast). Consistent with this observation, Δ*hpdA* conidia remain viable in macrophages. Plating the contents of lysed Δ*hpdA* infected macrophages at 37°C *in vitro* resulted in the growth of Δ*hpdA* colonies.

To assess if deletion of genes of the tyrosine catabolic cluster affects the phagocytosis of conidia by macrophages, conidia of the wildtype, Δ*hpdA*, Δ*hmgA*, Δ*hmgX* and Δ*maiA* strains were used to infect murine J774 macrophages and the macrophages examined 2 hours post-infection. No differences in phagocytosis were observed (average number of conidia per 100 macrophages: wildtype 76.7±4.70, Δ*hpdA* 67.3*±9*.*80*, Δ*hmgA* 70.0*±*9.90, Δ*hmgX* 75.0*±*6.70 and Δ*maiA* 79.3*±*8.20).

The wildtype, Δ*hpdA*, Δ*hmgA* and Δ*hmgX* strains were also used to infect human THP-1 macrophages. The Δ*hpdA*, Δ*hmgA* and Δ*hmgX* phenotypes in human macrophages were identical to those described for infection of murine macrophages (S6 Fig).

## Discussion

### The tyrosine catabolic cluster is subject to complex regulatory control

The expression of genes within the *P*. *marneffei* tyrosine catabolic gene cluster is subject to a complex network of regulatory control with four different levels of regulation; pathway specific regulation by the Zn(II)2-Cys6 binuclear transcription factor HmgR, cluster specific regulation by location, global regulation in response to nitrogen source by AreA and post-transcriptional feedback by pathway intermediates. The complex regulation of the tyrosine catabolism gene cluster reflects the multiple roles played by genes in this cluster, not only in adapting to the nutritional sources available but also the production of protective melanin. An additional, unique role for HpdA in *P*. *marneffei*, and probably other dimorphic fungi with an intracellular (host cell) phase, is also suggested.

Genes of the tyrosine catabolic cluster are regulated specifically in response to the presence of tyrosine by the Zn(II)2-Cys6 binuclear transcription factor HmgR encoded within the cluster. HmgR is required for full induction of these genes in the presence of tyrosine as the sole nitrogen source and deletion results in reduced and no growth on tyrosine as a sole nitrogen and carbon source, respectively. The orthologue in *A*. *fumigatus*, *hmgR*, is also required for tyrosine-induced expression of genes in the cluster, suggesting this protein plays a conserved role [5]. However, unlike *A*. *fumigatus*, the *P*. *marneffei* Δ*hmgR* mutation results in only a partial loss of tyrosine-induced expression, suggesting that an additional factor contributes to positively regulating expression of the tyrosine catabolic cluster in response to tyrosine. Zn(II)2-Cys6 binuclear transcription factors have been shown to positively regulate the expression of many nitrogen metabolic pathways in fungi [5, 34, 35, 36, 37]. Unexpectedly, deletion of *hmgR* in *P*. *marneffei* also resulted in partial derepression on ammonium suggesting that HmgR also has a negative role in regulating gene expression.

The genes of the tyrosine catabolic cluster are also under specific regulation by location. The expression of *mfpA*, the gene within the tyrosine metabolic cluster only in *P*. *marneffei* and *T*. *stipitatus* which does not appear to have a role in tyrosine catabolism, was induced on tyrosine at both 25°C and 37°C even though it does not share a promoter with another cluster gene. This suggests that any gene captured within this genomic region may consequently come under its regulation. Similar effects have been described for other gene clusters (for example *spoCI* in *A*. *nidulans*) [38]. In addition, the complementation strains which had the wildtype gene reintroduced at the *pyrG* or *niaD* loci, did not show complete restoration of wildtype growth on tyrosine and phenylalanine, also indicating that the position of the gene in the cluster is important for it’s regulation.

AreA is a positively-acting GATA-type transcription factor which regulates a large number of genes to effect nitrogen metabolite repression; the global response to limiting nitrogen conditions [30, 39]. We originally hypothesized that the additional factor contributing to positively regulating expression of the tyrosine catabolic cluster in response to tyrosine could be AreA. In *A*. *nidulans*, AreA remodels chromatin in cooperation with Zn(II)2-Cys6 binuclear transcription factors in response to specific nitrogen sources [36, 40]. However, deletion of *areA* in *P*. *marneffei* did not reduce induction of the tyrosine catabolic cluster genes in the presence of tyrosine but rather unexpectedly lead to a loss of repression in the presence of ammonium. This suggests that contrary to the paradigm, AreA is acting negatively on the tyrosine catabolic cluster genes under nitrogen repressing conditions. To our knowledge, only two examples exist of AreA acting as a negative regulator in fungi despite examples of a related human GATA factor acting as both an activator and repressor [41, 42, 43]. In *A*. *nidulans*, AreA negatively regulates the expression of *nadA*, an adenine deaminase encoding gene required for purine degradation, though unlike the *P*. *marneffei* tyrosine cluster genes, *nadA* is also expressed on ammonium [41]. There is only 4 bp between the unique UaY (positively acting Zn(II)2-Cys6 binuclear transcription factor) and AreA binding sites in the *nadA* promoter and AreA acts in part to negate UaY-mediated induction by directly competing with UaY binding [41]. However, the identification of potential AreA binding sites in the promoters of *P*. *marneffei* tyrosine catabolic cluster genes (HGATAR) shows that no predicted sites are in close proximity to the potential HmgR binding site predicted by RSAT analysis. AreA also acts as a repressor of genes required for arginine catabolism in *A*. *nidulans* [43]. Similar to the *P*. *marneffei* tyrosine gene cluster, genes required for arginine catabolism in *A*. *nidulans* are not expressed in ammonium, are induced by arginine and this induction is dependent on the ArcA Zn(II)2-Cys6 binuclear transcription factor [35]. Also similar to tyrosine, arginine can be utilized as both a nitrogen and carbon source. Under carbon repressing conditions (1% glucose) an *areA* loss of function mutant results in a loss of nitrogen metabolite repression of the arginine catabolism genes [43].

Tyrosine catabolism is also regulated by post-transcriptional feedback by pathway intermediates. Deletion of genes of the tyrosine catabolic cluster (*hpdA*, *hmgA*, *hmgX* and *maiA*) resulted in reduced growth on tyrosine as a sole nitrogen source. The proposed transamination reaction of tyrosine with α-ketoglutarate to produce glutamate as a nitrogen source occurs prior to the reactions catalysed by HpdA, HmgA and MaiA. This is indicative of a negative feedback loop regulating the catabolism of tyrosine in *P*. *marneffei*. This regulatory mechanism appears to be conserved as the *hmgR* and *hmgX* deletions in *A*. *fumigatus* also show reduced growth on tyrosine or phenylalanine as the sole nitrogen source [5].

### The role of tyrosine catabolism and pyomelanin production during pathogenic growth of yeast cells

Dimorphism is intricately linked to pathogenicity and therefore differentially expressed genes, particularly those that display yeast-specific expression, are likely to play a role in the establishment and progression of infection. Based on this hypothesis, yeast-specific genes in *P*. *marneffei* were identified using a microarray-based expression profiling [6]. This profiling revealed that *hpdA*, encoding the enzyme 4-hydroxyphenylpyruvate dioxygenase (HpdA) required during the catabolism of tyrosine, was induced specifically in the pathogenic yeast cell type at 37°C [6]. Intriguingly, chemical inhibition of HpdA activity using NTBC or deletion of *hpdA* resulted in a severe defect in the production of yeast cells during macrophage infection, suggesting that HpdA is required for *P*. *marneffei* pathogenicity. Therefore the question arose as to whether this was a result of reduced nutrient acquisition, reduced melanin protection against oxidative stress or a combination of both. Interestingly, chemical inhibition of HpdA resulted in more significant delay in conidial germination compared to deletion of *hpdA* (Figs. 1 and 9). This is likely due to the inhibition of the host 4-hydroxyphenylpyruvate dioxygenase, as well as that of the fungus, thus leading to a further decrease in available nutrients for growth.

Deletion of *hpdA*, as well as other cluster genes encoding enzymes required for tyrosine catabolism (*hmgA*, *hmgX* and *maiA*) and the gene encoding the regulatory transcription factor (*hmgR*) resulted in reduced growth on tyrosine as the sole nitrogen or carbon source at both 25°C and 37°C, showing that these genes are required for tyrosine catabolism and that *P*. *marneffei* can utilize tyrosine as both a nitrogen and carbon source for growth. However, unlike Δ*hpdA*, the Δ*hmgX*, Δ*maiA* and Δ*hmgR* strains did not show reduced yeast cell production in macrophages. The Δ*hmgA* mutant showed slightly reduced yeast cell production but this is likely to be attributed to reduced conidial germination due accumulation of homogentisate and consequent hypermelanization. This result suggests that nitrogen or carbon acquisition via tyrosine catabolism is not essential for the early stages of infection in macrophages and that the phenotype of the Δ*hpdA* mutant is not due to reduced nutrient acquisition. In addition, the lack of conidial germination in the Δ*hpdA* in macrophages is unlikely to be due to a toxic build-up of tyrosine catabolic intermediates, as the Δ*hmgA* mutant, which showed a greater reduction in growth than the Δ*hpdA* mutant due to toxicity *in vitro*, displayed only a mild reduction in yeast cell production *ex vivo*. Somewhat unexpectedly, Δ*hmgX* did not show the same phenotype as Δ*hpdA* during macrophage infection, despite the hypothesis that HmgX acts as an accessory protein to HpdA and the Δ*hpdA* and Δ*hmgX* strains showing an equivalent growth reduction on tyrosine as a sole nitrogen or carbon source at both 25°C and 37°C and an equivalent lack of pyomelanin production *in vitro*. It is possible that HmgX is only partially required for HpdA function under certain conditions. This hypothesis is supported by the reduced accumulation of toxic intermediates of the tyrosine catabolic pathway compared to Δ*hpdA* and Δ*hmgA*.

The *ex vivo* phenotype of the Δ*hpdA* mutant is unlikely to be a result of the inability to produce pyomelanin, as the Δ*hmgX* mutant, which like the Δ*hpdA* mutant cannot produce pyomelanin, produces wildtype levels of yeast cells in macrophages. It is possible that the Δ*hmgX* mutant can produce a colourless intermediate to the final pigmented pyomelanin which may be sufficient to afford protection in macrophages, however, the Δ*maiA* and Δ*hmgR* mutants, which displayed reduced pyomelanin production at 37°C, also displayed *ex vivo* growth indistinguishable from wildtype. Instead these results may suggest that HpdA has an additional role to tyrosine catabolism and pyomelanin formation in *P*. *marneffei* and may also explain why *hpdA* is highly constitutively expressed at 37°C, in contrast to all of the other genes in the tyrosine catabolic cluster. Therefore the question arises as to what this additional novel role of *hpdA* during *ex vivo* growth could be. It is possible that tyrosine catabolism, and specifically the step catalysed by HpdA, is acting as a developmental signal. The enzyme tyrosine aminotransferase, which catalyses the first step in tyrosine catabolism and which an orthologue is lacking in *P*. *marneffei*, has been shown to promote apoptosis and prevent cell proliferation during cancer development in humans [44]. In addition, inhibition of *hpd-*1 by RNAi in the nematode *Caenorhabditis elegans* extends lifespan and results in a developmental arrest at the dauer larval stage, which prevents the completion of development when environmental conditions are unfavourable [45]. In *C*. *elegans* the link between tyrosine catabolism and development is proposed to occur via the insulin-like *daf-2* signalling pathway [46]. Elevated tyrosine has inhibitory affects on DAF-2 signalling [47]. The DAF-2 receptor, orthologous to the insulin receptor in mammals, lies upstream of a phosphatidylinositol 3 signalling cascade which acts to phosphorylate the DAF-16 forkhead transcription factor preventing it’s entry into the nucleus [47]. Daf-16 positively regulates the expression of a number of genes regulating metabolism and lifespan including those encoding enzymes which protect against free radicals such as catalase-1 (*ctl-1*) and superoxide dismutase (*sod-3*) and 4-hydroxyphenylpyruvate dioxygenase (*hpd-1*) [45, 47]. The *P*. *marneffei* genome encodes a *daf-16* homologue (PMAA_055700) and the *hpdA* promoter contains a putative DAF-16 binding site (ATGTTTGA) which is conserved in the *Aspergilli* (consensus WTGTTTVV), suggesting that this regulatory mechanism may be conserved in fungi.

Further elucidation of the role played by *hpdA* during *ex vivo* growth will clearly provide insight into the progression of conidia into yeast cells during intracellular pathogenic growth of *P*. *marneffei* and opens up an exciting new avenue of investigation.

## Materials and Methods

### Molecular techniques and plasmid construction

*P*. *marneffei* genomic DNA was isolated as previously described [48]. Southern and northern blotting was performed with Amersham Hybond N+ membrane using [a-^32^P]dATP labeled probe hybridization using standard methods [49].

Sequences of primers are provided in S1 Table. A PCR product encompassing *P*. *marneffei hpdA* (PMAA_031950) was generated with primers AA48 and AA49 and cloned into pGemT Easy to generate pAM7072. To make the deletion construct, pKB7717, an *EcoRI/SalI* 5’ PCR product generated with MM70 and MM71 was cloned into *EcoRI/SalI* pBluescript II SK^+^. A *BamHI/SpeI* 3’ PCR product generated with MM72 and MM73 was then cloned into the *BamHI/SpeI* sites and subsequently a *BamHI/EcoRI pyrG* ^*b*^ fragment from pAB4626 was cloned into the *BamHI/EcoRI* sites. The complementation construct, pKB7682, was generated by cloning a *HindIII/SacI* fragment from pAM7072 into pLS7804 (*pyrG* targeting pBluescript II SK^+^). *hmgA* (PMAA_031960) was PCR amplified using primers MM75 and MM76 and cloned into pGemT Easy to generate pKB7723. The deletion construct, pKB7724, was generated by replacing the *BglII/StuI* fragment from pKB7723 with *BamHI/EcoRV pyrG* ^*b*^ from pAB4626. The complementation construct, pKB7791, was generated cloning a *BamHI/XhoI* digested PCR product generated using OO78 and OO79 into *BamHI/XhoI* pLS7804. *maiA* (PMAA_032000) was cloned by PCR amplification with primers PP18 and PP19 and cloned into pGemT Easy to generate pKB7786. The deletion construct, pKB7805, was generated by replacing the *BglII*/*XhoI* fragment from pKB7786 with a *BamHI/XhoI* fragment from pAB4626. The *maiA* complementation construct, pKB858, was generated by cloning a *PstI/EcoRV* fragment from pKB7786 into *PstII/EcoICRI* pHB7615 (*niaD* targeting pBluescript SK^*+*^). *hypW* (PMAA_031870) was cloned by PCR amplification with primers KK37 and KK38 and cloned into pGemT Easy to generate pSM7504. The deletion construct, pSM7546, was generated by ligating an inverse PCR product generated using primers KK71 and KK72 to *EcoRV/SmaI pyrG* ^*b*^ from pAB4626. Likewise, *hmgX* (PMAA_031980) was cloned by PCR amplification with primers KK39 and KK40 and cloned into pGemT Easy to generate pSM7505. The deletion construct, pSM7651, was generated by ligating an inverse PCR product generated using primers KK73 and KK74 to *EcoRV/SmaI pyrG* ^*b*^ from pAB4626. The *hypW* and *hmgX* complementation construct, pKB7801, was generated by cloning a *SpeI/StuI* fragment from pSM7505 into *XbaI/EcoICRI* pLS7804. The complementation construct containing *hypW* and a truncated *hmgX* allele, pKB7940, was generated by cloning a *PstI/HindIII* fragment from pSM7505 into *PstI/HindIII* pHB7615. The regulatory gene *hmgR* (PMAA_032020) was cloned by ligating the PCR product generated using primers LL13 and LL14 into *EcoRV* digested pBluescript II SK^+^, to generate pSH7541. The deletion construct, pSH7578, was generated by cloning an *EcoRV/SmaI pyrG* ^*b*^ fragment from pAB4626 into an inverse PCR product of pSH7541 with primers LL56 and LL57. The complementation construct, pKB7683, was generated by cloning in the *KpnI/SpeI* fragment from pSH7541 into *KpnI/XbaI* pLS7804. *mfpA* (PMAA_032010) was PCR amplified using primers QQ50 and QQ52 and cloned into pGemT Easy to generate pLS7828. The deletion construct, pLS7852, was generated using a Gateway method as described in [50] using the primers QQ57 and QQ58. *hpdB* (PMAA_089170) was amplified using primers PP14 and PP15 and cloned into pGemT Easy to generate pLS7785. The deletion construct, pLS7845, was generated using the Gateway method using the primers QQ59 and QQ60. *wA* (PMAA_082120) was amplified using primers HH45 and HH46 and cloned into pGemT Easy to generate pSJ7351. The deletion construct, pHW7445, was generated by replacing the *BglII/EcoRV* fragment of pSJ7351 with the *BamHI/EcoRV pyrG* ^*b*^ fragment from pAB4626.

### Fungal strains and media

Transformation was performed using the previously described protoplast method [48]. Strains used in this study are listed in Table 2. The Δ*hpdA pyrG* ^*+*^ strain (G827) was generated by transformation of strain G832 (*pkuA TK barA niaD1 pyrG1*) with linearised pKB7717 which removes sequences from -11 to +1517 of *hpdA* and selecting for pyrG^+^. The Δ*hmgA*::*pyrG* ^*+*^ (G823), Δ*hypW*::*pyrG* ^*+*^ (G854), Δ*hmgX*::*pyrG* ^*+*^ (G856), Δ*maiA*::*pyrG* ^*+*^ (G895), Δ*mfpA*::*pyrG* ^*+*^ (G946) and Δ*hpdB* strains were generated by transforming strain G816 (Δ*ligD niaD1 pyrG1*) with linearised deletion constructs pKB7724 (which removes from +138 to +717 of *hmgA*), pSM7546 (which removes from +5 to +1145 of *hypW*), pSM7651 (which removes from -53 to +929 of *hmgX*), pKB7805 (which removes from +42 to +249 of *maiA*) and pLS7852 (removes from -22 to +2741 of *mfpA*), pLS7845 (which removes from -39 to +1127 of *hpdB*) and pSH7578 (which removes from -278 to +2601 of *hmgR*), and selecting for pyrG^+^ or glufosinate resistance. The Δ*wA*::*pyrG* ^*+*^ (G748) strain was generated by transforming strain G147 (*niaD1 pyrG1*) with pHW7445 and selecting for pyrG^+^. Deletion was confirmed by genomic Southern blot analysis. *pyrG* ^-^ strains (Table 2) were generated by plating on medium containing 1 mg mL^-1^ 5-fluoroorotic acid (5-FOA) supplemented with 10 mM NH_4_SO_4_ and 5 mM uracil to select for the loss of the *pyrG* marker. These strains are unable to grow in the absence of 5 mM uracil. The deletion mutants were complemented by transformation of *pyrG* ^-^ strains with pKB7682 (*hpdA*), pKB7791 (*hmgA*), pKB7800 (*maiA*), pKB7801 (*hypW* ^*+*^ *hmgX* ^*+*^), pKB7940 (*hypW* ^*+*^ *hmgX* ^*trun*^), pKB7801 (*hmgX*), pKB7683 (*hmgR*) and selecting for *pyrG* ^*+*^ or transformation of G895 with pKB7858 (*maiA*) and selecting for *niaD* ^+^. Integration of the complementation constructs at *pyrG* or *niaD* was confirmed by Southern blot analysis of genomic DNA.

**Table 2:** Strains used in this study.

Number	Strain name	Genotype	Source
G320	FRR2161	Wildtype	
G147	SPM4	*niaD pyrG*	[48]
G809	Δ*ligD*::*pyrG* ^*+*^	Δ*ligD*::*pyrG* ^*+*^ *niaD pyrG*	[50]
G816	Δ*ligD pyrG* ^-^	Δ*ligD niaD pyrG*	[50]
G944	Δ*ligD pyrG* ^*t*^	Δ*ligD niaD pyrG* [*pyrG* ^*t*^ *SK* ^*+*^]	This study
G832	*pkuA tkb pyrG* ^-^	*pkuA*::*hv-tk*::*barA niaD pyrG*	H. Weerasinghe and A. Andrianopoulos
G945	*pkuA tkb pyrG* ^*t*^	*pkuA*::*hv-tk*::*barA niaD pyrG* [*pyrG* ^*t*^ *SK* ^*+*^]	This study
G827	Δ*hpdA pyrG* ^*+*^	*pkuA*::*hv-tk*::*barA niaD pyrG* Δ*hpdA*::*pyrG* ^*+*^	This study
G866	Δ*hpdA pyrG* ^-^	*pkuA*::*hv-tk*::*barA niaD pyrG* Δ*hpdA*	This study
G901	Δ*hpdA hpdA* ^*+*^	*pkuA hv-tk barA niaD pyrG* Δ*hpdA* [*pyrG* ^*t*^ *hpdA* ^*+*^]	This study
G823	Δ*hmgA pyrG* ^*+*^	Δ*ligD niaD pyrG* Δ*hmgA*::*pyrG* ^*+*^	This study
G862	Δ*hmgA pyrG* ^-^	Δ*ligD niaD pyrG* Δ*hmgA*	This study
G938	Δ*hmgA hmgA* ^*+*^	Δ*ligD niaD pyrG* Δ*hmgA* [*pyrG* ^*t*^ *hmgA* ^*+*^]	This study
G895	Δ*maiA pyrG* ^*+*^	Δ*ligD niaD pyrG* Δ*maiA*::*pyrG* ^*+*^	This study
G951	Δ*maiA maiA* ^*+*^	Δ*ligD pyrG* Δ*maiA* [*niaD* ^*t*^ *maiA* ^*+*^]	This study
G854	Δ*hypW pyrG* ^*+*^	Δ*ligD niaD pyrG* Δ*hypW*::*pyrG* ^*+*^	This study
G858	Δ*hypW pyrG* ^-^	Δ*ligD niaD pyrG* Δ*hypW*	This study
G897	Δ*hypW hypW* ^*+*^ *hmgX* ^*+*^	Δ*ligD niaD pyrG* Δ*hypW* [*pyrG* ^*t*^ *hypW* ^*+*^ *hmgX* ^*+*^]	This study
G986	Δ*hypW hypW* ^*+*^ *hmgX* ^*trun*^	Δ*ligD niaD pyrG* Δ*hypW* [*pyrG* ^*t*^ *hypW* ^*+*^ *hmgX* ^*trun*^]	This study
G856	Δ*hmgX pyrG* ^*+*^	Δ*ligD niaD pyrG* Δ*hmgX*::*pyrG* ^*+*^	This study
G860	Δ*hmgX pyrG* ^-^	Δ*ligD niaD pyrG* Δ*hmgX*	This study
G899	Δ*hmgX hmgX* ^*+*^	Δ*ligD niaD pyrG* Δ*hmgX* [*pyrG* ^*t*^ *hmgX* ^*+*^]	This study
G825	Δ*hmgR pyrG* ^*+*^	Δ*ligD niaD pyrG* Δ*hmgR*::*pyrG* ^*+*^	This study
G864	Δ*hmgR pyrG* ^-^	Δ*ligD niaD pyrG* Δ*hmgR*	This study
G867	Δ*hmgR hmgR* ^*+*^	Δ*ligD niaD pyrG* Δ*hmgR* [*pyrG* ^*t*^ *hmgR* ^*+*^]	This study
G946	Δ*mfpA pyrG* ^*+*^	Δ*ligD niaD pyrG*.Δ*mfpA*::*pyrG* ^*+*^	This study
G748	Δ*wA pyrG* ^*+*^	Δ*wA*::*pyrG* ^*+*^ *niaD pyrG*	This study
G939	Δ*hpdB bar* ^*R*^ *pyrG* ^-^	Δ*ligD niaD pyrG* Δ*hpdB*::*barA* ^*R*^	This study
G911	Δ*areA*	Δ*areA*::*ptrA* ^*R*^	T. Woodward and A. Andrianopoulos

To test growth on tyrosine or phenylalanine as a sole nitrogen source, strains were grown at 25°C and 37°C on *A*. *nidulans* minimal medium (ANM) supplemented with 1% glucose and 10 mM ammonium sulphate ((NH_4_)_2_SO_4_), 10 mM phenylalanine, or 10 mM tyrosine [51,52]. To test growth on tyrosine or phenylalanine as a sole carbon source, strains were grown at 25°C and 37°C for 14 days on *A*. *nidulans* minimal medium (ANM) supplemented with 10 mM ammonium sulphate ((NH_4_)_2_SO_4_) and 50 mM phenylalanine or 10 mM tyrosine [51,52]. NTBC (2-(2-nitro-4-trifluoromethylbenzoyl)-cyclohexane-1, 3-dione) was added for HpdA inhibition experiments at a final concentration of 400μg mL^-1^. To assess pyomelanin production, strains were grown at 37°C for 14 days on 1% glucose *A*. *nidulans* minimal medium (ANM) supplemented with either 10 mM ammonium sulphate (NH_4_SO_4_), 10 mM tyrosine, both 10 mM NH_4_SO_4_ and 10 mM tyrosine or both 10 mM tyrosine and 10 mM alanine. Strains were also grown at 37°C on BHI for 5 days. The growth of the Δ*hmgR* strain was assessed after 14 days on 10 mM alanine, arginine, asparagine, cysteine, glutamate, glycine, histidine, isoleucine, leucine, lysine, methionine, phenylalanine, proline, serine, threonine, tryptophan, tyrosine or valine as the sole nitrogen source at both 25°C and 37°C. To test the effect of accumulating toxic tyrosine catabolism intermediates on growth, the wildtype (WT) and Δ*hpdA*, Δ*hmgA* and Δ*hmgX* strains were grown on carbon-free medium containing 10mM Gaba and either 10mM sorbitol, 1% lactose, 10mM NaAc or 10mM proline with or without 10mM tyrosine for 14 days at 25°C.

L-DOPA medium was prepared by making a 50 mL dH_2_0 solution containing 0.5 g L-asparagine, 0.5 g glucose, 1.5 g KH_2_PO_4_, 0.125 g MgSO_4_-7H_2_0 and 100 mg L-DOPA and adjusting the pH to 5.6. Subsequently, 0.5 mg thiamine-HCL and 0.5 mL of 1M biotin solution were added and the solution filter sterilized. This solution was added to 500 mL of sterilized 2% agar solution. Strains were grown at 37°C for 14 days.

Oxidative stress was tested by plating on ANM + Supps medium supplemented with 10 mM tyrosine and 0 mM, 0.25 mM, 0.5 mM, 0.75 mM and 1 mM of H_2_O_2_. Plates were inoculated with 10 μL drops of 10 fold serial dilutions of a 1x 10^7^ conidia mL^-1^ suspension and were incubated at 37°C for 14 days.

### Purification of melanin particles

The wildtype and Δ*hpdA* mutant were grown on ANM + Supps medium supplemented with 10 mM tyrosine for 14 days at 37°C. Cells were suspended in 0.1M sodium citrate and 1M sorbitol, pH 5.5 plus 10mg/mL lytic enzyme overnight at 30°C. Cells were pelleted and resuspended in 4M guanidine thiocyanate solution and incubated at room temperature overnight. Cells were then washed in PBS, resuspended in 6.6M HCL and boiled for 1.5 hours. The remaining particles (none were visible for the Δ*hpdA* mutant) were centrifuged and the pellets washed 3x in PBS and resuspended (in PBS).

### Macrophage assay

J774 murine macrophages (1 x 10^5^) or THP-1 human macrophages were seeded into each well of a 6 well microtitre tray containing one sterile coverslip and 2 mL of complete Dulbecco’s Modified Eagle Medium for J774 (complete DMEM: DMEM, 10% fetal bovine serum, 8 mM L-glutamine and penicillin-streptomycin) or 2 mL of RPMI (complete RPMI: RPMI, 10% fetal bovine serum, 2 mM L-glutamine and penicillin-streptomycin) for THP-1. Macrophages were incubated at 37°C for 24 hours before activation with 0.1 μg mL^-1^ lipopolysaccharide (LPS) from *E*. *coli* (Sigma) and THP-1 macrophages differentiated with phorbol 12-myristate 13-acetate. Macrophages were incubated a further 24 hours at 37°C, washed in phosphate buffered saline (PBS) and 2 mL of complete DMEM or RPMI medium containing 1 x 10^6^ conidia was added. A control lacking conidia was also performed. Macrophages were incubated for 2 hours at 37°C (to allow conidia to be engulfed), washed once in PBS (to remove free conidia) and incubated a further 24 or 48 hours at 37°C. Macrophages were fixed in 4% paraformaldehyde and stained with 1 mg mL^-1^ fluorescent brightener 28 (calcofluor—CAL) to observe fungal cell walls. Mounted coverslips were examined using differential interference contrast (DIC) and epifluorescence optics for cell wall staining and viewed on a Reichart Jung Polyvar II microscope. Images were captured using a SPOT CCD camera (Diagnostic Instruments Inc) and processed in Adobe Photoshop^TM^. The numbers of ungerminated conidia, germlings or yeast cells were recorded in a population of approximately 100 in three independent experiments. Mean and standard error of the mean values were calculated using GraphPad Prism3. 400μg mL^-1^ of NTBC was added at the time of infection for HpdA inhibition experiments.

### Expression analysis

Liquid cultures of FRR2161 (wildtype), Δ*hmgR* and 2206*areA* strains grown for 2 days at 25°C in ANM plus 10 mM (NH_4_)_2_SO_4_ and 6 days at 37°C in BHI were used to inoculate ANM plus 10 mM (NH_4_)_2_SO_4_, 10 mM alanine or, 10 mM tyrosine as the sole nitrogen source at 25°C and 37°C and RNA was isolated after 4 hours. Liquid cultures of FRR2161 (wildtype), Δ*hmgR* and Δ*areA* strains grown for 2 days at 25°C in ANM plus 10 mM (NH_4_)_2_SO_4_ were used to inoculate ANM plus 10 mM tyrosine or 10 mM tyrosine and 10 mM (NH_4_)_2_SO_4_ at 25°C and RNA was isolated after 4 hours. 2 day 25°C liquid cultures of Δ*hypW* grown in ANM with 10 mM (NH_4_)_2_SO_4_, was used to inoculate ANM with 10 mM (NH_4_)_2_SO_4_ or 10 mM tyrosine as the sole nitrogen source at 25°C and RNA was isolated after 4 hours. RNA was extracted using TRIzol Reagent (Invitrogen) and a MP FastPrep-24 bead beater according to the manufacturer’s instructions. At least two biological repeats were performed. Illumina RNA sequencing was performed on RNA isolated from liquid cultures of FRR2161 (wildtype) grown for 6 days at 37°C in BHI. RNA was DNAase treated (Promega) prior to RT PCR analysis. For every gene in this study, 3 increasing cycle numbers were used on two biological repeats to ensure the product was not saturated and the result was representative. *H3* (histone H3) was used as a loading control. Expression was determined by RT PCR using primers CC53 and CC54 (*hpdA*), DD13 and DD14 (*hmgA*), MM64 and MM65 (*hmgX*), MM32 and MM33 (*hypW*), DD19 and DD20 (*fahA*), DD17 and DD18 (*maiA*), II94 and II95 (*mfpA*), II88 and II89 (*hmgR*), and GG4 and GG5 (*H3*).

## Supporting Information

S1 Fig Complementation of the growth phenotypes of deletion strains of genes in the tyrosine catabolic cluster. Growth of the wildtype (WT) and the Δ*hpdA hpdA* ^*+*^, Δ*hmgA hmgA* ^*+*^, Δ*hmgX hmgX* ^*+*^, Δ*maiA maiA* ^*+*^ and Δ*hmgR hmgR* ^*+*^ complemented strains (A) and Δ*mfpA* and Δ*hpdB* strains (B) on carbon and nitrogen free medium (C and N free), on ammonium as the sole nitrogen source (gluc NH_4_), on phenylalanine as the sole nitrogen source (gluc phe), on phenylalanine as the sole carbon source (phe NH_4_), on tyrosine as the sole nitrogen source (gluc tyr) or on tyrosine as the sole carbon source (tyr NH_4_) after 14 days at 25°C.
(TIF)

S2 Fig Growth toxicity due to the accumulation of tyrosine catabolic intermediates. Growth after 14 days at 25°C of the wildtype (WT), Δ*hpdA*, Δ*hmgA* and Δ*hmgX* strains on carbon-free medium containing 10mM GABA and either 10mM sorbitol, 1% lactose, 10mM acetate or 10mM proline with or without 10mM tyrosine.
(TIF)

S3 Fig HypW is not required for tyrosine catabolism at 25°C. A. RNA was isolated from wildtype (*hmgR* ^+^) and Δ*hmgR* strains grown in liquid culture for 2 days at 25°C or 6 days at 37°C and transferred into media containing ammonium (NH_4_) or tyrosine (Tyr) as the sole nitrogen source at 25°C or 37°C for 4 hours. Expression of *hypW* (PMAA_031970) was detected by RT PCR. B. Illumina RNA sequencing reads over the 2.8kb genomic region encompassing *hmgX* and *hypW*. The *hmgX* and *hypW* gene annotations are shown in blue and coding sequence (CDS) annotations in yellow. Reads spanning the *hypW* gene annotation do not cover the predicted start site and are present within the predicted intron, suggesting the CDS annotation is incorrect. C. Growth of the Δ*hypW* mutant compared to Δ*hypW hypW* ^*+*^ *hmgX* ^*+*^ and Δ*hypW hypW* ^*+*^ *hmgX* ^*trun*^ on carbon and nitrogen free medium (C and N free), on ammonium as the sole nitrogen source (gluc NH_4_), on phenylalanine as the sole nitrogen source (gluc phe), on phenylalanine as the sole carbon source (phe NH_4_), on tyrosine as the sole nitrogen source (gluc tyr) or on tyrosine as the sole carbon source (tyr NH_4_) after 14 days at 25°C (A) or 37°C (B). D. RNA from wildtype (WT) and Δ*hypW* strains grown in liquid culture for 2 days at 25°C and transferred into media containing ammonium (NH_4_) or tyrosine (Tyr) as the sole nitrogen source at 25°C for 4 hours. Expression of *hmgX* and a *H3* loading control was detected by RT PCR. Expression of *hmgX* is decreased in the Δ*hypW* strain.
(TIF)

S4 Fig Reintroduction of the wildtype gene complements the growth phenotypes of deletion strains at 37°C. Growth after 14 days at 37°C of the wildtype (WT) and the Δ*hpdA hpdA* ^*+*^, Δ*hmgA hmgA* ^*+*^, Δ*hmgX hmgX* ^*+*^, Δ*maiA maiA* ^*+*^ and Δ*hmgR hmgR* ^*+*^ complemented strains (A) and Δ*mfpA* and Δ*hpdB* (B) on carbon and nitrogen free medium (C and N free), on ammonium as the sole nitrogen source (gluc NH_4_), on phenylalanine as the sole nitrogen source (gluc phe), on phenylalanine as the sole carbon source (phe NH_4_), on tyrosine as the sole nitrogen source (gluc tyr) or on tyrosine as the sole carbon source (tyr NH_4_).
(TIF)

S5 Fig Deletion of *hpdA* results in no melanin production when grown on tyrosine at 37°C and reduces melanisation on BHI medium. A. Melanin particles isolated from wildtype and the Δ*hpdA* mutant grown on medium containing tyrosine as the sole nitrogen source at 37°C after 14 days. In contrast to wildtype, no melanin particles were observed in the Δ*hpdA* mutant after boiling in acid. B. Growth of the wildtype, Δ*hpdA*, Δ*hpdA hpdA* ^*+*^, Δ*hmgA*, Δ*hmgA hmgA* ^+^, Δ*hmgX*, Δ*hmgX hmgX* ^*+*^, Δ*maiA*, Δ*maiA maiA* ^*+*^, Δ*hmgR*, Δ*hmgR hmgR* ^*+*^, Δ*mfpA*, Δ*wA* and Δ*areA* strains on BHI medium at 37°C after 5 days. Deletion of genes of the tyrosine catabolic cluster reduces melanisation on BHI medium at 37°C. C. *Wildtype and* Δ*areA* grown on L-DOPA medium for 14 days at 37°C.
(TIF)

S6 Fig ex vivo growth in human macrophages. Conidia of the wildtype, Δ*hpdA*, Δ*hmgA* and Δ*hmgX* were used to infect THP-1 human macrophages and growth was assessed 24 hours post-infection. After 24 hours, macrophages infected with wildtype conidia contain numerous yeast cells dividing by fission. In contrast, ungerminated conidia were predominately observed in macrophages infected with conidia of the Δ*hpdA* mutant 24 hours post-infection. The Δ*hmgA* strain showed a small increase in the number of ungerminated conidia and a small decrease in the number of yeast cells. The Δ*hmgX* mutant was indistinguishable from wildtype.
(TIF)

S1 Table Oligonucleotides used in this study. (DOC)
