# Supplementary figures and images for: Intracellular Growth Is Dependent on Tyrosine Catabolism in the Dimorphic Fungal Pathogen Penicillium marneffei
Source: PLoS Pathog. 2015 Mar 26;11(3):e1004790. doi: 10.1371/journal.ppat.1004790 (PMC4374905; doi:10.1371/journal.ppat.1004790)

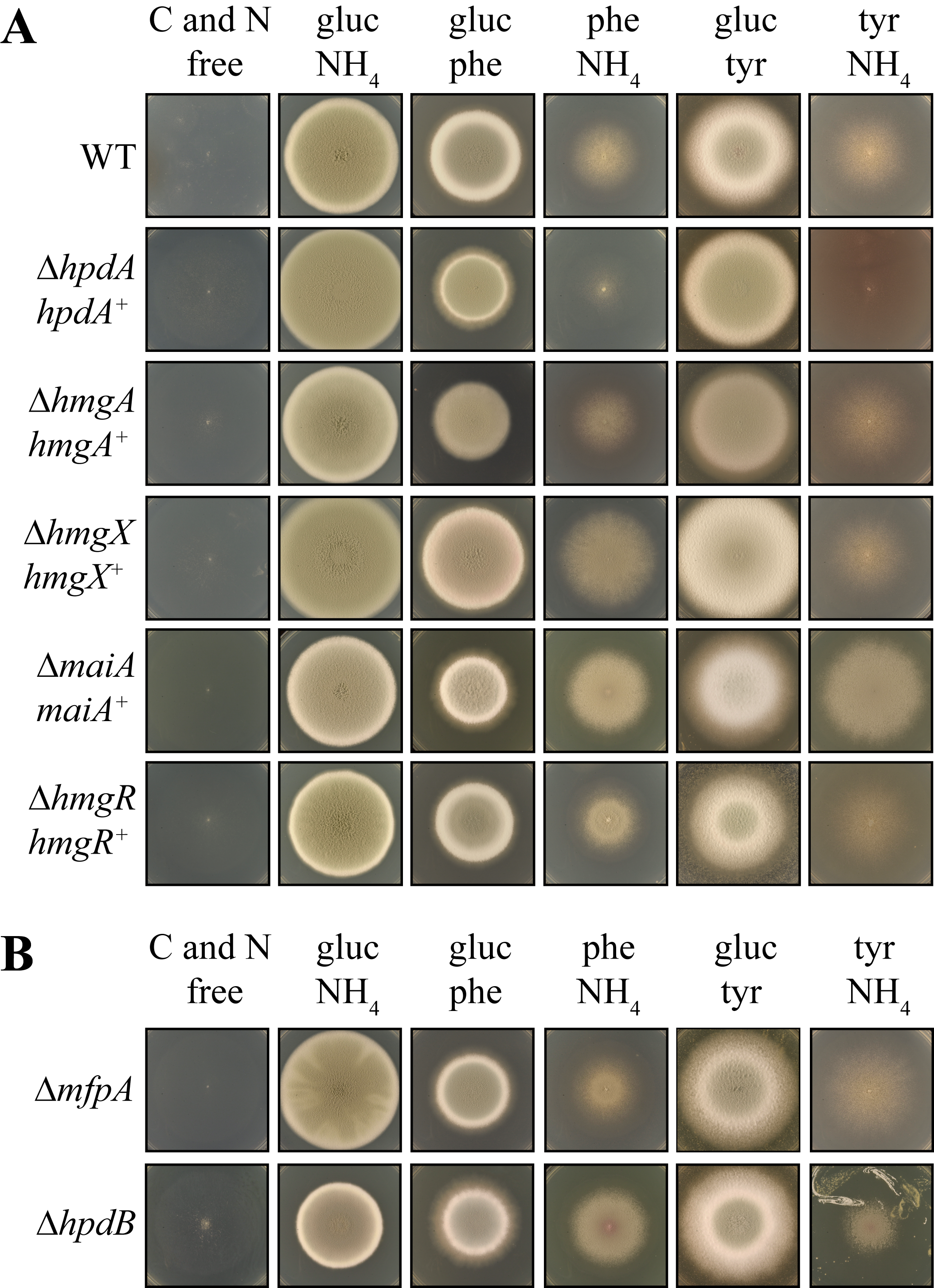

Supplement: S1 Fig — Growth of the wildtype (WT) and the ΔhpdA hpdA +, ΔhmgA hmgA +, ΔhmgX hmgX +, ΔmaiA maiA + and ΔhmgR hmgR + complemented strains (A) and ΔmfpA and ΔhpdB strains (B) on carbon and nitrogen free medium (C and N free), on ammonium as the sole nitrogen source (gluc NH4), on phenylalanine as the sole nitrogen source (gluc phe), on phenylalanine as the sole carbon source (phe NH4), on tyrosine as the sole nitrogen source (gluc tyr) or on tyrosine as the sole carbon source (tyr NH4) after 14 days at 25°C. (TIF) [file ppat.1004790.s001.tif]

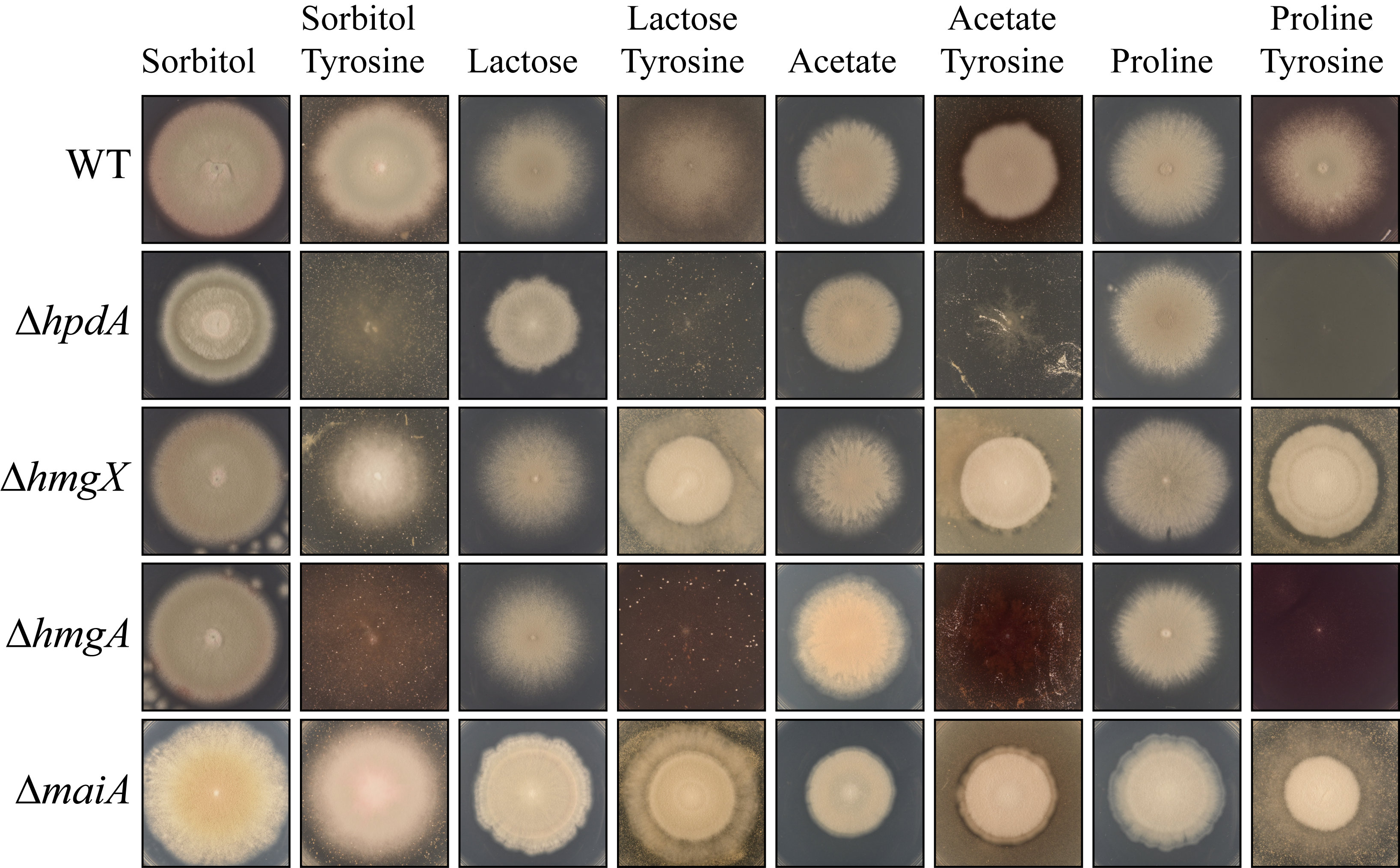

Supplement: S2 Fig — Growth after 14 days at 25°C of the wildtype (WT), ΔhpdA, ΔhmgA and ΔhmgX strains on carbon-free medium containing 10mM GABA and either 10mM sorbitol, 1% lactose, 10mM acetate or 10mM proline with or without 10mM tyrosine. (TIF) [file ppat.1004790.s002.tif]

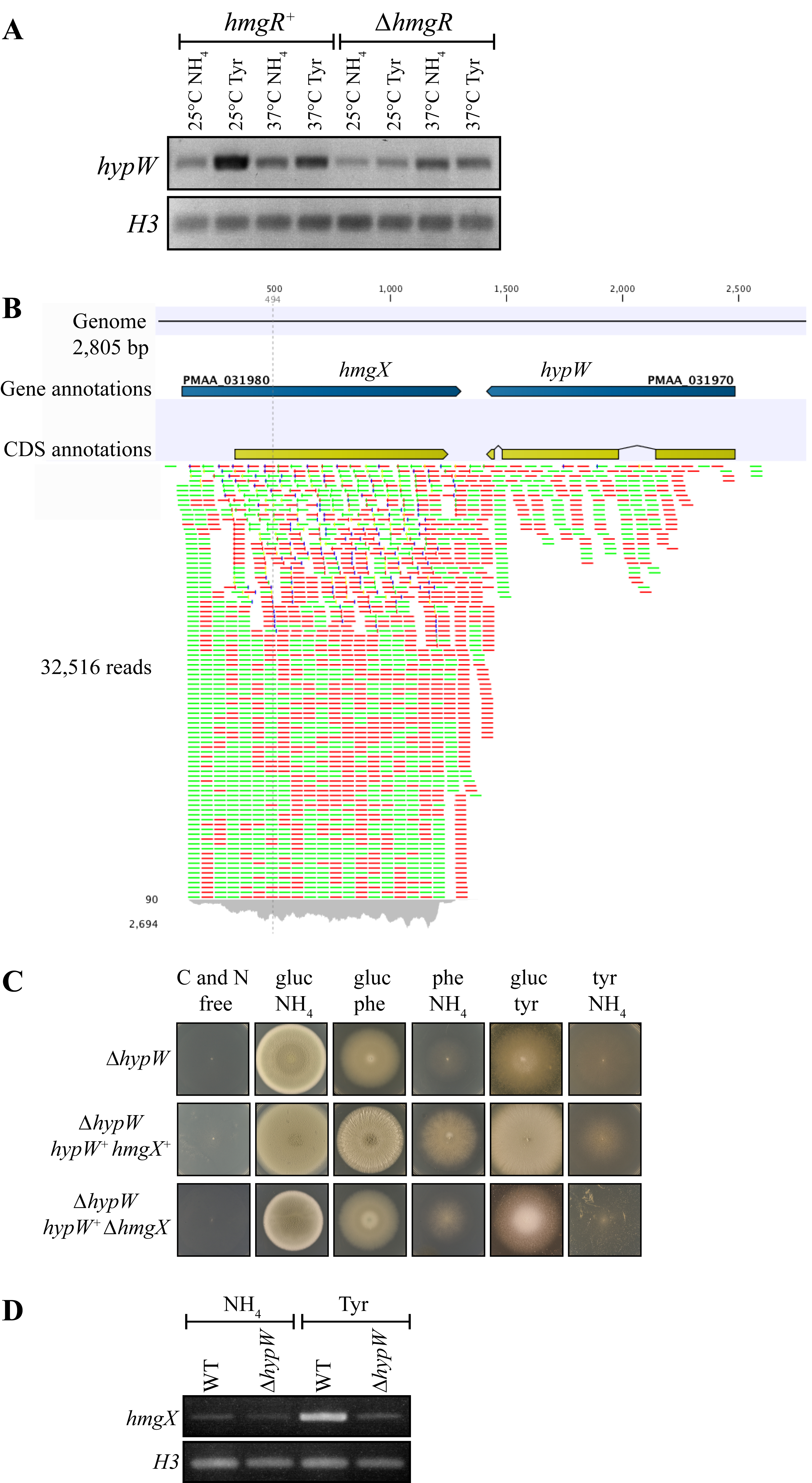

Supplement: S3 Fig — A. RNA was isolated from wildtype (hmgR +) and ΔhmgR strains grown in liquid culture for 2 days at 25°C or 6 days at 37°C and transferred into media containing ammonium (NH4) or tyrosine (Tyr) as the sole nitrogen source at 25°C or 37°C for 4 hours. Expression of hypW (PMAA_031970) was detected by RT PCR. B. Illumina RNA sequencing reads over the 2.8kb genomic region encompassing hmgX and hypW. The hmgX and hypW gene annotations are shown in blue and coding sequence (CDS) annotations in yellow. Reads spanning the hypW gene annotation do not cover the predicted start site and are present within the predicted intron, suggesting the CDS annotation is incorrect. C. Growth of the ΔhypW mutant compared to ΔhypW hypW + hmgX + and ΔhypW hypW + hmgX trun on carbon and nitrogen free medium (C and N free), on ammonium as the sole nitrogen source (gluc NH4), on phenylalanine as the sole nitrogen source (gluc phe), on phenylalanine as the sole carbon source (phe NH4), on tyrosine as the sole nitrogen source (gluc tyr) or on tyrosine as the sole carbon source (tyr NH4) after 14 days at 25°C (A) or 37°C (B). D. RNA from wildtype (WT) and ΔhypW strains grown in liquid culture for 2 days at 25°C and transferred into media containing ammonium (NH4) or tyrosine (Tyr) as the sole nitrogen source at 25°C for 4 hours. Expression of hmgX and a H3 loading control was detected by RT PCR. Expression of hmgX is decreased in the ΔhypW strain. (TIF) [file ppat.1004790.s003.tif]

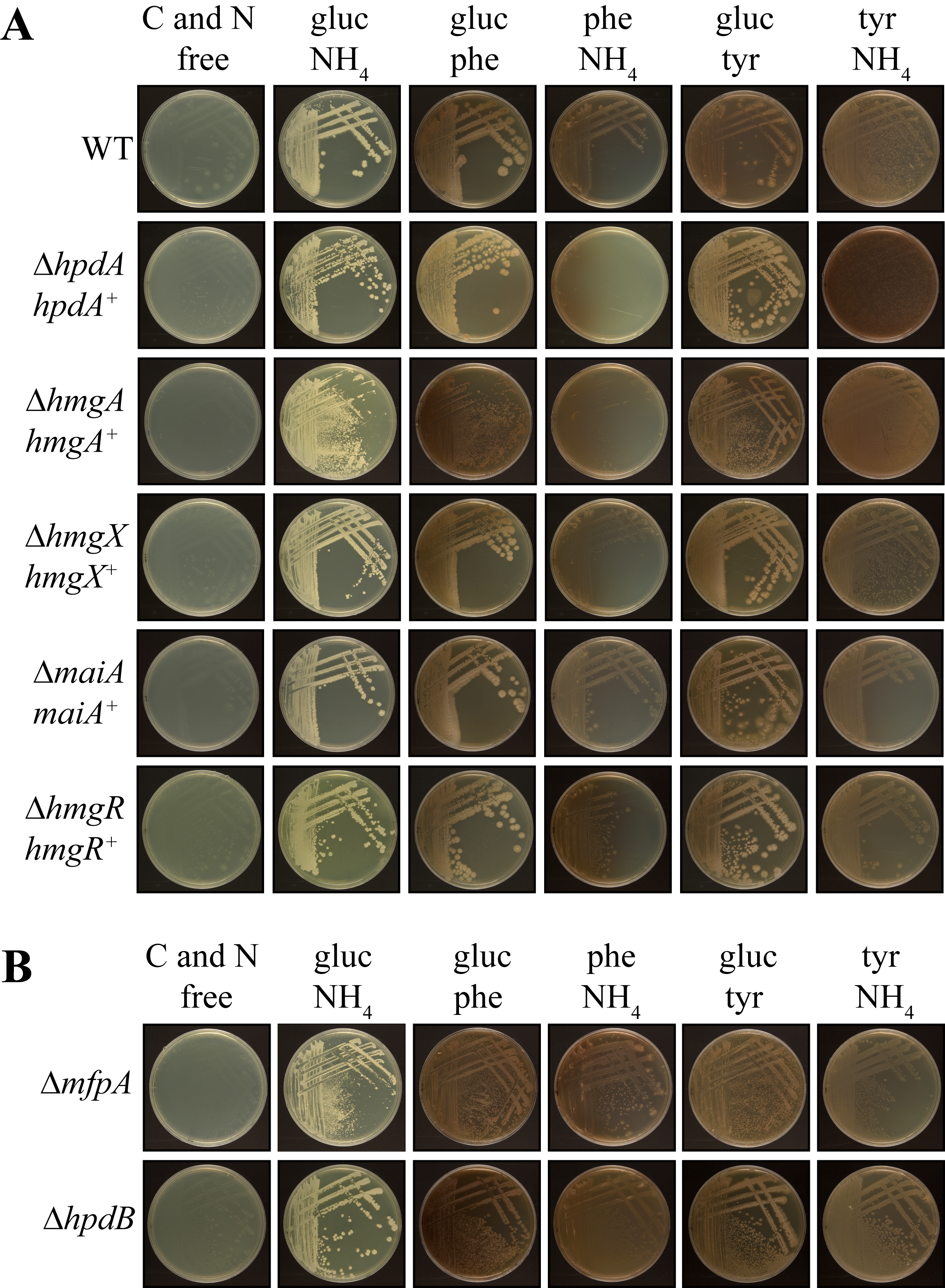

Supplement: S4 Fig — Growth after 14 days at 37°C of the wildtype (WT) and the ΔhpdA hpdA +, ΔhmgA hmgA +, ΔhmgX hmgX +, ΔmaiA maiA + and ΔhmgR hmgR + complemented strains (A) and ΔmfpA and ΔhpdB (B) on carbon and nitrogen free medium (C and N free), on ammonium as the sole nitrogen source (gluc NH4), on phenylalanine as the sole nitrogen source (gluc phe), on phenylalanine as the sole carbon source (phe NH4), on tyrosine as the sole nitrogen source (gluc tyr) or on tyrosine as the sole carbon source (tyr NH4). (TIF) [file ppat.1004790.s004.tif]

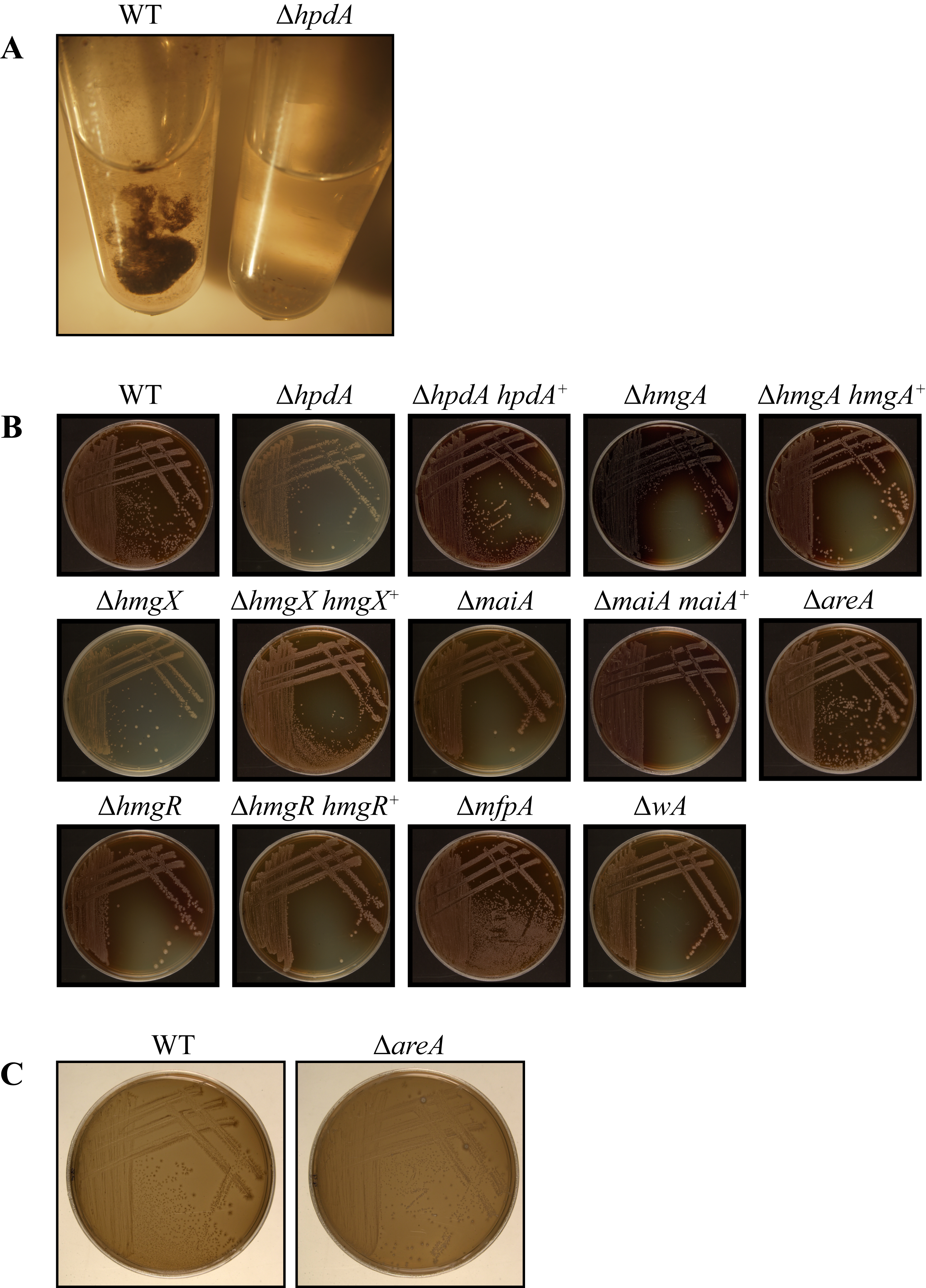

Supplement: S5 Fig — A. Melanin particles isolated from wildtype and the ΔhpdA mutant grown on medium containing tyrosine as the sole nitrogen source at 37°C after 14 days. In contrast to wildtype, no melanin particles were observed in the ΔhpdA mutant after boiling in acid. B. Growth of the wildtype, ΔhpdA, ΔhpdA hpdA +, ΔhmgA, ΔhmgA hmgA +, ΔhmgX, ΔhmgX hmgX +, ΔmaiA, ΔmaiA maiA +, ΔhmgR, ΔhmgR hmgR +, ΔmfpA, ΔwA and ΔareA strains on BHI medium at 37°C after 5 days. Deletion of genes of the tyrosine catabolic cluster reduces melanisation on BHI medium at 37°C. C. Wildtype and ΔareA grown on L-DOPA medium for 14 days at 37°C. (TIF) [file ppat.1004790.s005.tif]

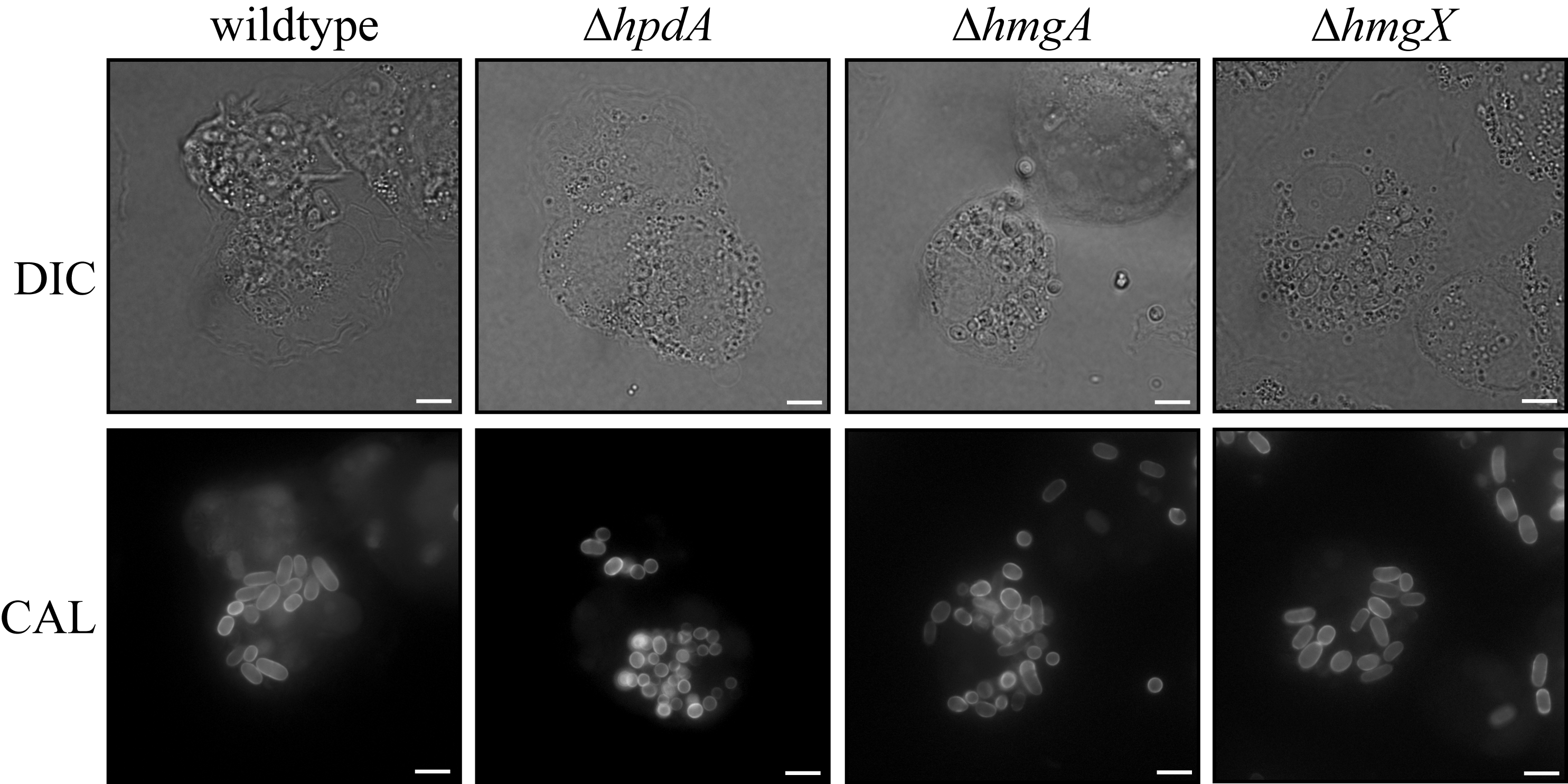

Supplement: S6 Fig — Conidia of the wildtype, ΔhpdA, ΔhmgA and ΔhmgX were used to infect THP-1 human macrophages and growth was assessed 24 hours post-infection. After 24 hours, macrophages infected with wildtype conidia contain numerous yeast cells dividing by fission. In contrast, ungerminated conidia were predominately observed in macrophages infected with conidia of the ΔhpdA mutant 24 hours post-infection. The ΔhmgA strain showed a small increase in the number of ungerminated conidia and a small decrease in the number of yeast cells. The ΔhmgX mutant was indistinguishable from wildtype. (TIF) [file ppat.1004790.s006.tif]
