# Supplementary material for: Intracellular Growth Is Dependent on Tyrosine Catabolism in the Dimorphic Fungal Pathogen Penicillium marneffei
Source: PLoS Pathog. 2015 Mar 26;11(3):e1004790. doi: 10.1371/journal.ppat.1004790 (PMC4374905; doi:10.1371/journal.ppat.1004790)
Supplement: S1 Table — (DOC) [file ppat.1004790.s007.doc]

**Table S1. Oligonucleotides used in this study**

| Gene | Number | Sequence |
| --- | --- | --- |
| *hpdA* | AA48 | GGAGAACTTAGTGACGGGA |
|  | AA49 | AACTATCAGCAAGCCGTG |
|  | MM70 | TTCTCGTCTGGGTCGTTG |
|  | MM71 | ACCTGCAGATTGTCGTGTGTACTGGC |
|  | MM72 | GGATCCTTTGTCGTTTTCTCTATC |
|  | MM73 | GTACTAGTGGTGGTCGTAGGAGATTG |
|  | CC53 | CGGTGCCGTACACAGGTATT |
|  | CC54 | TTGATTTCAGGGCGGAGTAG |
| *hmgA* | MM75 | GCGCACACCTTCTCCATC |
|  | MM76 | TATCCGGGAAGTTAGGGC |
|  | OO78 | CCGGATCCACCCTCTTCAAATCTACC |
|  | OO79 | AACTCGAGACCAAGGATTCTATTCAG |
|  | DD13 | ATTAGAGCAACACGAAAC |
|  | DD14 | AGGATTACCATTTCACAG |
| *maiA* | PP18 | CAGGCAACAAACTCGGTC |
|  | PP19 | GAACGCGGGGAGAAAGAG |
|  | DD17 | CGCTACTATTCCATCCAT |
|  | DD18 | GGTAGGTATCCGCTGGGT |
| *hypW* | KK37 | TTATTCCGAGGGGTGTCAAG |
|  | KK38 | CCGTTGAAGCCAGGTAAATG |
|  | KK71 | GCCATCTGGTCCGAAGAAT |
|  | KK72 | CGTGTAATAGTCACACAATG |
|  | MM32 | TGCCAACGCATTTATTCTGA |
|  | MM33 | CACCTCCGAGACGATTTGAC |
| *hmgX* | KK39 | GTTGAAGCCGCAGAAGGTAG |
|  | KK40 | CCGAAACGACAACCTCAAAT |
|  | KK73 | TCAAGCGATGCTATTTGGTTT |
|  | KK74 | GCAATTTTGAATTTATTGTGGAA |
|  | MM64 | AGTTGGTTGGGACCTCCTCT |
|  | MM65 | GCTATATTCCATCCCGCAAA |
| *hmgR* | LL13 | CAAAAGTATCAAACAACCTC |
|  | LL14 | TACTGAGTCTGGAAATGAAA |
|  | LL56 | CTCGTAGTAGATGAAGATTA |
|  | LL57 | CTTATTGTCGCTCAGGGTTA |
|  | II88 | CACCATGCGCAAGATGTC |
|  | II89 | GATCCGAGAAGCATCCAG |
| *fahA* | DD19 | GTGCGAAACTCAGTGGTA |
|  | DD20 | CTCGCAAACAGAAATCGT |
| *hpdB* | PP14 | ATGAGAATCCTTGCACTGGG |
|  | PP15 | TGACACAACCTCTTAGCCCC |
|  | QQ59 | GGGGACCCAGCTTTCTTGTACAAAGTGGTTATGCGTTCAACTGAGATTC |
|  | QQ60 | GGGGAGCCTGCTTTTTTGTACAAACTTGTTTGTGTAAAATAGGTCTAAC |
| *mfpA* | QQ52 | TGCCAAATCCTCAAGAAAG |
|  | QQ57 | GGGGACCCAGCTTTCTTGTACAAAGTGGTTATCTAGCGCAAGTATTT |
|  | QQ58 | GGGGAGCCTGCTTTTTTGTACAAACTTGTAGATTTCAAGAGAGCGAC |
|  | QQ50 | AACTCGAGATCCCCCTACATTTGCACC |
|  | II94 | CTGATTTTCGAAGTTCCG |
|  | II95 | GCCAAAGCCAACATAATC |
| *H3* | GG4 | GGTGTCAAGAAGCCTCATCGT |
|  | GG5 | GATGGCGGAGGACTGGAA |
| *wA* | HH45 | GTCATGCAGGAAAGGGTCAT |
|  | HH46 | GCACCGGTCGATACTTGAAT |
